# Phragmalin-Type Limonoids from the Fruits of *Chukrasia tabularis* and Their Anti-Inflammatory Activity

**DOI:** 10.3390/molecules28135136

**Published:** 2023-06-30

**Authors:** Shujun Dai, Yuzhen Wu, Xiujuan Xin, Faliang An

**Affiliations:** 1 State Key Laboratory of Bioreactor Engineering, East China University of Science and Technology, 130 Meilong Road, Shanghai 200237, China; 20000862@mail.ecust.edu.cn (S.D.); 18721589937@163.com (Y.W.); xinxj@ecust.edu.cn (X.X.); 2 Marine Biomedical Science and Technology Innovation Platform of Lin-gang Special Area, No. 4, Lane 218, Haiji Sixth Road, Shanghai 201306, China

**Keywords:** *Chukrasia tabularis*, meliaceae, limonoid, anti-inflammatory

## Abstract

Phytochemical investigation on the fruits of *C. tabularis* led to the isolation of five new phragmalin-type limonoids (**1**–**5**) and four known ones (**6**–**9**). The structures of the new compounds **1**–**5**, named chuktabamalins A–E, were elucidated via spectroscopic techniques (HRESIMS, 1D and 2D NMR) and were comparable with the literature data of known compounds. In addition, new compounds were evaluated for in vitro anti-inflammatory activity. Compounds **1**, **2**, **3** and **5** showed moderate anti-inflammatory activity with IC_50_ values of 21.72 ± 2.79, 23.29 ± 1.00, 47.08 ± 3.47 and 66.67 ± 2.89 μM, respectively.

## 1. Introduction

Limonoids as the major secondary metabolites from Meliaceae family, are well-known for their abundance, structural diversity and a wide range of antifeedant, antimalarial, antimicrobial, cytotoxic and growth-regulating activities [1,2,3,4,5,6,7,8]. The genus *Chukrasia* (Meliaceae) comprises only *Chukrasia tabularis* A. Juss and *Chukrasia tabularis* var. *velutina*, which are mainly distributed in the tropical areas of Asia, such as India, Malaysia and southern China [9]. In folk medicine, the root bark of *C. tabularis* has been used for dispelling wind and heat from the body by the peoples in the tropical areas of Asia [10]. Phragmalin-type limonoids are the characteristic components of *Chukrasia* genus, such as normal phragmalins and their orthoesters [11], 13/14/18-cyclopropanyl phragmalin-type limonoids [11,12], C(15)-acyl 16-norphragmalins [12], C(15)-acyl phragmalins [13,14], 16-norphragmalins [15], 19-dinorphragmalins [16] and 16,19-dinorphragmalins [17].

**Figure 1:**
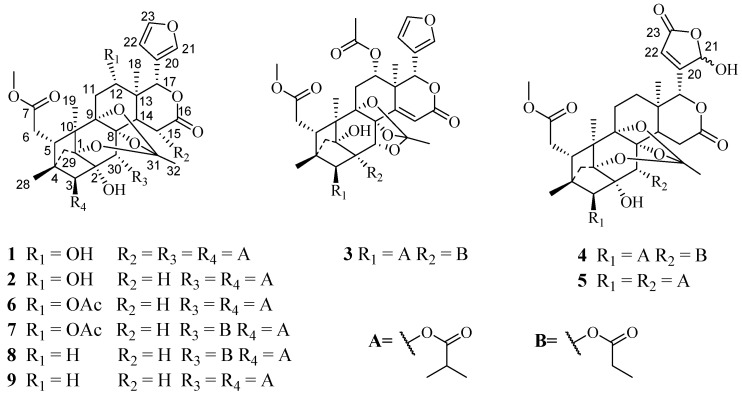
The chemical structures of compounds **1**–**9**.

The further investigation of limonoids from this plant, provided five new phragmalin-type limonoids, chuktabamalins A-E (**1**–**5**) (Figure 1) and four known compounds (**6**–**9**), which were isolated from the EtOH extracts of the fruits of *Chukrasia tabularis*. Herein, the isolation and structure elucidation of the new compounds as well as their anti-inflammatory evaluation are reported.

## 2. Results and Discussion

Chuktabamalin A (**1**) was obtained as a white amorphous powder, and a molecular formula of C_41_H_54_O_16_ was deduced from a HRESIMS peak at *m*/*z* 825.3284 [M + Na]^+^ (calculated for C_41_H_54_O_16_Na^+^ 825.3304), indicating the presence of 15 degrees of unsaturation. The strong IR absorptions at 1743 and 3448 cm^−1^ implied the presence of ester carbonyl and hydroxyl groups, respectively. The ^1^H-NMR (Table 1) exhibited signals for ten methyl groups (*δ*_H_ 0.92 (3H, s), 1.08 (3H, s), 1.14 (3H, d, *J* = 6.9 Hz), 1.15 (3H, s), 1.19 (3H, d, *J* = 6.5 Hz), 1.20 (3H, d, *J* = 6.9 Hz), 1.22 (3H, d, *J* = 7.0 Hz), 1.25 (3H, d, *J* = 7.0 Hz), 1.26 (3H, d, *J* = 6.5 Hz) and 1.61 (3H, s)), one methoxy group (*δ*_H_ 3.69 (3H, s)) and five oxymethine protons (*δ*_H_ 4.03 (1H, dd, *J* = 10.6, 5.9 Hz), 4.68 (1H, s), 5.61 (1H, s), 6.01 (1H, s) and 6.55 (1H, d, *J* = 3.2 Hz)), as well as three aromatic methine protons (*δ*_H_ 6.61 (1H, s), 7.53 (1H, s) and 7.69 (1H, s)). The ^13^C-NMR displayed the corresponding carbons, in addition to three methylenes, five oxygenated methines and fourteen quaternary carbons (3-furanyl aromatic quaternary carbon, five carbonyls and five oxygenated), as supported by DEPT-135 and HSQC experiments. The further analysis of ^1^H- and ^13^C-NMR (Table 1 and Table 2), the structural framework of a phragmalin-type limonoid with a 1,8,9-*ortho* ester was suggested for **1**, including the presence of a typical *β*-substituted furan ring (*δ*_H_ 6.61, 7.53, 7.69; *δ*_C_ 109.3, 121.3, 141.6 and 144.5), six membered *δ*-lactone (*δ*_H_ 2.17, 5.61 and 6.55; *δ*_C_ 42.4, 52.8, 64.0, 76.6 and 168.4), three tertiary methyls (*δ*_H_ 0.92, 1.08 and 1.15; *δ*_C_ 12.8, 15.0 and 16.5) and five ester carbonyls (*δ*_C_ 168.4, 173.6, 175.1, 175.8 and 176.6). Based on the extensive analysis of 2D NMR (^1^H-^1^H COSY and HMBC) spectra, it is inferred that there are three isobutyryloxy groups present.

**Figure 2:**
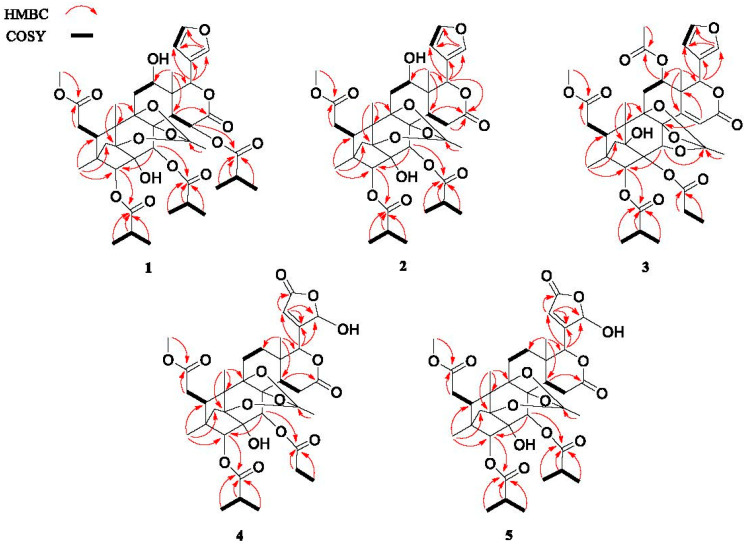
Selected ^1^H-^1^H COSY and HMBC correlations of **1**–**5**.

The NMR spectroscopic data indicated that **1** had a similar skeleton to that of phragmalin 3, 30-di-isobutyrate (**9**), except for the different substitution patterns at C-12 and C-30. A hydroxy group was assignable to C-12 (*δ*_C_ 66.5), which was supported by the ^1^H-^1^H COSY correlation of H-11/H-12, as well as the HMBC correlations of H-18/C-12 (Figure 2). The ^1^H-^1^H COSY correlations of H-2″/H-3″ and H-2″/H-4″, as well as the HMBC correlations of H-2″, H-3″, H-4″ and H-30/C-1″ indicated that the isobutyryloxy moiety was attached to C-30 (Figure 2). Therefore, the planar structure of 1 was elucidated as indicated.

**Figure 3:**
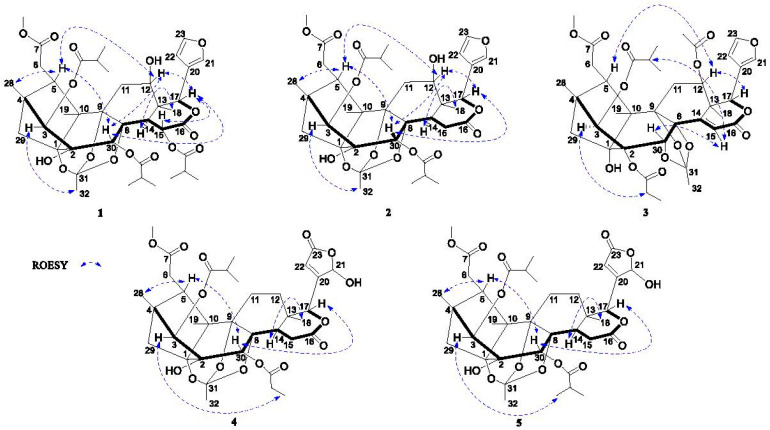
Key ROESY correlations of **1**–**5**.

The relative configuration of 1 was established using the ROESY spectrum (Figure 3), in which the correlations of H-12/H-5, H-12/H-17, H-17/H-30, H-15/H-17 and H-5/H-28, indicated that these protons were arbitrarily assigned as the *β*-orientation. Moreover, the ROESY correlations of H-32/H-3 and H-14/H-18 were observed, which demonstrated that these protons had an *α*-orientation. Accordingly, the structure of compound **1** was established and named chuktabamalin A.

Chuktabamalin B (**2**), a white amorphous powder, had a molecular formula of C_37_H_48_O_14_ on the basis of its prominent positive HRESIMS ion peak at *m*/*z* 739.2968 ([M + Na]^+^, C_37_H_48_O_14_Na^+^ calculated as 739.2936). A comparison between the NMR data (Table 1 and Table 2) of **1** and **2** showed that the isobutyryloxy group at C-15 in **1** was absent in **2**, which was supported by the ^1^H-^1^H COSY correlations of H-14/H-15, together with the HMBC correlations from H-18/C-14, H-14/C-16, H-15/C-16 and H-17/C-16. The relative configuration of **2**, supported by the analysis of a ROESY experiment, was the same as those of **1**. Therefore, the structure of **2** was established as shown in Figure 1.

Chuktabamalin C (**3**), a white amorphous powder, displayed a peak for [M + Na]^+^ at *m*/*z* 765.2707 (calculated for C_38_H_46_O_15_Na^+^ as 765.2729) in the HRESIMS spectrum, requires 16 degrees of unsaturation. The ^13^C NMR and DEPT spectroscopic data of **3** displayed 38 carbon signals, which was in an agreement with the molecular formula, including nine methyl (one methoxy group), four methylene, ten methine (five oxygenated and four olefinic) and fifteen quaternary carbons (five carbonyl, two olefinic and five oxygenated). These data were similar to those of xyloccensin T [18], suggesting that compound **3** was also an 8,9,30-phragmalin *ortho* ester. The major differences between **3** and xyloccensin T were the substituents at C-2 and C-3. In the detailed analysis of the 1D (^1^H NMR, ^13^C NMR and DEPT) and 2D NMR data (^1^H-^1^H COSY, HSQC and HMBC), one isopropyloxy group and one propionyloxy group were indicated. The isopropyloxy moiety was attached to C-3, which was supported by the ^1^H-^1^H COSY correlations of H-2′/H-3′ and H-2′/H-4′, as well as the HMBC correlations of H-2′, H-3′, H-4′ and H-3/C-1′(Figure 2). The obvious downfield signal at *δ*_C_ 83.5 (C-2) in ^13^C NMR data (Table 2) suggested that the propionyloxy group was attached to C-2.

The relative configuration of **3** was deduced by the analysis of its ROESY spectrum. As shown in Figure 2, the observed ROESY correlations of H-5/H-12, H-12/H-17, H-15/H-30 and H-15/H-2′ indicated that the isopropyloxy and acetoxyl were *β*-oriented, whereas the correlations of H-3/H-2′ demonstrated that the propionyloxy was α-oriented. Therefore, the structure of compound **3** was finally established, as shown in Figure 1.

Chuktabumalin D (**4**) was obtained as a white, amorphous powder with a molecular formula of C_36_H_46_O_15_, as established at the basis of the prominent HRESIMS ion peak at *m*/*z* 741.2718 ([M + Na]^+^, calculated as 741.2729). The ^1^H-NMR (Table 1) and ^13^C-NMR (Table 2) along with the HSQC data of **4** revealed the presence of one methoxy group, five carbonyl carbons, seven methyls, six methylenes and eight methines with four oxygenated and nine quarternary carbons. These data were similar to those of phragmalin 3-isobutyrate 30-propionate (**8**), suggesting that compound **4** was also an 1,8,9-phragmalin *ortho* ester. The main differences between them were the presence of a lactone carbonyl (*δ*_C_ 169.1) signal and an acetal methine (*δ*_H_ 6.18; *δ*_C_ 97.5) signal and the absence of two olefinic methine signals in **4** compared to phragmalin 3-isobutyrate 30-propionate (**8**). HMBC correlations between H-22/C-20, C-21, C-23, H-17/C-20, H-17/C-21 and H-17/C-22 indicated that a *β*-furyl ring moiety located at C-17 in phragmalin 3-isobutyrate 30-propionate was replaced by a 21-hydroxy-20(22)-en-21,23-*γ*-lactone moiety in **4**. The remaining substructure was determined to be the same as phragmalin 3-isobutyrate 30-propionate based on the 2D NMR data, as shown in Figure 2. The nearly identical chemical shifts and *J*-values suggested that compound **4** and phragmalin 3-isobutyrate 30-propionate shared the same relative configuration. This deduction was confirmed via ROESY correlations (Figure 3). Therefore, the structure of **4**, named chuktabumalin D, was established as shown in Figure 1.

Chuktabumalin E (**5**) was isolated as a white, amorphous powder. Its molecular formula of C_37_H_48_O_15_ was established by HRESIMS (*m*/*z* 755.2871, calculated as 755.2885). The NMR data of **5** were similar to those of compound **4** except for the different substitution at C-30. The isopropyloxy group was confirmed by the ^1^H-^1^H COSY correlations of H-2″/H-3″ and H-2″/H-4″, as well as the HMBC correlations of H-2″, H-3″, H-4″ and H-30/C-1″, which indicated that the isopropyloxy moiety was attached to C-30 (Figure 2). Through the extensive analysis of the 1D and 2D NMR data, the structure of **5** was established, as shown in Figure 1.

Four known compounds, 12α-acetoxyphragmalin 3,30-di-isobutyrate (**6**), 12α-acetoxyphragmalin 3-isobutyrate 30-propionate (**7**), phragmalin 3-isobutyrate 30-propionate (**8**) and phragmalin 3,30-di-isobutyrate (**9**) [19], were identified through the comparison of their spectroscopic data with the literature data.

Compounds **1**–**5** were assessed for inhibitory effects on lipopolysaccharide-induced nitric oxide production in RAW264.7 cells in vitro. Compounds **1**, **2**, **3** and **5** showed moderate anti-inflammatory activity (Table 3).

## 3. Materials and Methods

### 3.1. General Experimental Procedures

Optical rotations were determined using a JASCO P-1020 polarimeter (JASCO Corporation, Tokyo, Japan). The NMR spectra were recorded in CDCl_3_ on a Bruker AVIII-500 spectrometer (Bruker, Bremen, Germany), using TMS as an internal standard. High-resolution electrospray ionization (HRESIMS) was recorded on an Agilent 6529B Q-TOF instrument (Agilent Technologies, Santa Clara, CA, USA). Column chromatography was performed using Silica gel (200–300 mesh, Qingdao Marine Chemical Inc., Qingdao, China), a Sephadex LH-20 (Merck, Darmstadt, Germany) and ODS (50 µm, YMC, Kyoto, Japan). TLC was performed using silica gel GF_254_ (Marine Chemical Industry Factory, Qingdao, China) and was detected by spraying with 5% H_2_SO_4_−EtOH.

### 3.2. Plant Material

The fruits of *C. tabularis* were collected in Danzhou, Hainan Province, People’s Republic of China, in November 2020. The plant was identified by Prof. Zhengfu Dai of the Institute of Tropical Bioscience and Biotechnology, Chinese Academy of Tropical Agricultural Sciences. A voucher specimen (NO. 20201120) was deposited at State Key Laboratory of Bioreactor Engineering laboratory.

### 3.3. Extraction and Isolation

The air-dried and powdered fruits of *C. tabularis* (15.2 kg) were extracted three times with 95% EtOH at room temperature to obtain a crude extract. The extract was then dissolved in water and partitioned with EtOAc, *n*-BuOH to give two parts. The EtOAc portion (2.6 kg) was subjected to a silica gel column and eluted with petroleum ether (PE)-EtOAc (from 10:1 to 0:1) to yield fifteen major fractions (Fr.1–Fr.15). Fr.8 (35.0 g) was applied to a vacuum liquid chromatography on silica gel and eluted with a gradient of CHCl_3_-MeOH (from 200:1 to 20:1) to give four parts (Fr.8A–Fr.8E). Fr.8B (15.0 g) was applied to an ODS gel column and eluted with MeOH-H_2_O (gradient from 40:60 to 100:0) to give four fractions (Fr.8B1–Fr.8B4). Fr.8B2 (602.0 mg) was applied to a silica gel column (PE-CHCl_3_-isopropyl alcohol, from 5:5:0.1 to 5:5:0.2) to obtain five subfractions (Fr.8B2A–Fr.8B2E), and then Fr.8B2B (120.0 mg) was subjected to a silica gel column (PE-acetone, 10:1) to yield **8** (9.0 mg). By the same purification procedures, Fr.8B2D yielded **5** (2.1 mg). Fr.8C (4.5 g) was separated via a silica gel column and eluted with PE–CHCl_3_–isopropyl alcohol (5:5:0.1 to 5:5:0.5) to obtain five fractions, namely Fr.8C1–Fr.8C5; then Fr.8C2 (220.0 mg) was separated on a silica gel column and eluted with PE-EtOAc (7:1) to obtain **9** (2.3 mg). Fr.8C4 (810.0 mg) was chromatographed on a silica gel column (PE-CHCl_3_-isopropyl alcohol, 5:5:0.1) to afford **2** (6.0 mg). Fr.9 (31.0 g) was separated on an ODS gel column and eluted with MeOH–H_2_O (gradient from 40:60 to 100:0) to give ten fractions, namelyFr.9A–Fr.4J, and then Fr.9C (1.8 g) was subjected to a silica gel column (PE–CHCl_3_–isopropyl alcohol, from 5:5:0.1 to 5:5:0.3) to yield **4** (11.2 mg). Fr.9E (2.8 g) was subjected to a silica gel column (PE–acetone, 9:1) to yield **6** (4.5 mg) and **7** (13.5 mg). Using the same purification procedures, Fr.9I yielded **1** (3.0 mg) and **3** (8.0 mg).

*Chuktabumalin A* (**1**): white, amorphous powder; [*α*]${}_{D}^{26}$ = −54 (*c* 0.10, MeOH); IR (KBr) *ν*_max_ 3448, 2965, 1743, 1632, 1465, 1388, 1149 cm^−1^; ^1^H and ^13^C NMR data (Figures S1–S6): Table 1 and Table 2; HRESIMS *m*/*z* 825.3284 [M + Na]^+^ (calculated for C_41_H_54_O_16_Na^+^ as 825.3304, Figure S7).
*Chuktabumalin B* (**2**): white, amorphous powder; [*α*]${}_{D}^{26}$ = +42 (*c* 0.10, MeOH); IR (KBr) *ν*_max_ 3447, 2928, 1744, 1631, 1456, 1266, 1103 cm^−1^; ^1^H and ^13^C NMR data (Figures S8–S13): Table 1 and Table 2; HRESIMS *m*/*z* 739.2968 [M + Na]^+^ (calculated for C_37_H_48_O_14_Na^+^ as 739.2936, Figure S14).
*Chuktabumalin C* (**3**): white, amorphous powder; [*α*]${}_{D}^{26}$ = +50 (*c* 0.10, MeOH); IR (KBr) *ν*_max_ 3446, 2927, 1729, 1631, 1462, 1382, 1237 cm^−1^; ^1^H and ^13^C NMR data (Figures S15–S20): Table 1 and Table 2; HRESIMS *m*/*z* 765.2707 [M + Na]^+^ (calculated for C_38_H_46_O_15_Na^+^ as 765.2729, Figure S21)
*Chuktabumalin D* (**4**): white, amorphous powder; [*α*]${}_{D}^{26}$ = −52 (*c* 0.12, MeOH); IR (KBr) *ν*_max_ 3448, 2963, 1744, 1630, 1464, 1387, 1152 cm^−1^; ^1^H and ^13^C NMR data (Figures S22–S27): Table 1 and Table 2; HRESIMS *m*/*z* 741.2718 [M + Na]^+^ (calculated for C_36_H_46_O_15_Na^+^ as 741.2729, Figure S28).
*Chuktabumalin E* (**5**): white, amorphous powder; [*α*]${}_{D}^{26}$ = −29 (*c* 0.12, MeOH); IR (KBr) *ν*_max_ 3449, 2964, 1743, 1631, 1387, 1150 cm^−1^; ^1^H and ^13^C NMR data (Figures S29–S34): Table 1 and Table 2; HRESIMS *m*/*z* 755.2871 [M + Na]^+^ (calculated for C_37_H_48_O_15_Na^+^ as 755.2885, Figure S28).

### 3.4. Anti-Inflammatory Activity

The anti-inflammatory activities of some compounds were evaluated for inhibitory activities against lipopolysaccharide (LPS)-induced nitricoxide (NO) production in macrophage (RAW264.7) cell lines. RAW 264.7 cells (5.0 × 10^4^ cells/mL) were incubated in 96-well plates with culture medium and incubated overnight at 37 °C in over 90% humidified atmosphere with 5% CO_2_. The cells were treated with compounds for 1 h and stimulated with LPS (500 ng/mL) for another 24 h. The NO inhibitory activity was determined via a Griess reaction. Briefly, the cell culture supernatant (100 μL) was mixed with an identical volume of Griess reagent, and the absorbance was recorded at 540 nm utilizing a plate reader. Quercetin was used as a positive control in the assays.

## 4. Conclusions

Compounds **1**–**9** were characterized as chuktabumalin A (**1**), chuktabumalin B (**2**), chuktabumalin C (**3**), chuktabumalin D (**4**), chuktabumalin E (**5**), 12α-acetoxyphragmalin 3,30-di-isobutyrate (**6**), 12α-acetoxyphragmalin 3-isobutyrate 30-propionate (**7**), phragmalin 3-isobutyrate 30-propionate (**8**) and phragmalin 3,30-di-isobutyrate (**9**), respectively. Compounds **1**–**5** were structurally determined to be new phragmalin-type limonoids. Compounds **1**, **2**, **3** and **5** showed moderate anti-inflammatory activity with IC_50_ values of 21.72 ± 2.79, 23.29 ± 1.01, 47.08 ± 3.47 and 66.67 ± 2.89 μM, respectively.

## Figures and Tables

**Table 1:** ^1^H NMR (500 MHz) data of compounds **1**–**5** (CDCl_3_, *δ*_H_ in ppm, *J* in Hz).

Proton	1	2	3	4	5
3	4.68 (s)	5.06 (s)	5.25 (s)	4.61 (s)	4.67 (s)
5	2.95 (t, 5.9)	2.26 (m)	2.16 (d, 10.0)	2.83 (d, 9.0)	2.86 (d, 9.2)
6	2.41 (dd, 11.6, 5.9)	2.43 (dd, 16.1, 10.3)	3.11 (d, 16.5)	2.53 (dd, 15.8, 9.7)	2.57 (m)
		2.30 (dd, 16.1, 1.7)	2.28 (m)	2.27 (m)	2.26 (dd, 15.9, 1.4)
11a	1.95 (d, 4.4)	2.51 (dd, 13.4, 5.2)	2.25 (m)	2.17 (m)	2.17 (d, 14.1)
11b	1.93 (s)	1.80 (d, 13.4)	1.99 (m)	1.70 (m)	1.72 (m)
12a	4.03 (dd, 10.6, 5.9)	3.81 (d, 5.0)	4.80 (dd, 13.4, 3.9)	1.54 (d, 12.5)	1.52 (d, 13.9)
12b				1.83 (d, 14.3)	1.90 (d, 10.8)
14	2.17 (d, 3.5)	2.87 (dd, 16.0, 3.7)		2.05 (d, 10.4)	2.06 (d, 10.0)
15a	6.55 (d, 3.2)	3.55 (dd, 14.6, 3.8)	6.69 (s)	3.23 (d, 20.0)	3.26 (d, 20.3)
15b		3.05 (t, 15.3)		2.66 (dd, 20.0, 10.6)	2.67 (dd, 20.2, 10.6)
17	5.61 (s)	4.99 (s)	5.98 (s)	5.57 (s)	5.64 (d, 1.4)
18	1.08 (s)	1.17 (s)	1.59 (s)	1.09 (s)	1.08 (s)
19	1.15 (s)	1.35 (s)	1.30 (s)	1.14 (s)	1.15 (s)
21	7.69 (s)	7.39 (s)	7.42 (s)	6.18 (s)	6.19 (s)
22	6.61 (s)	6.44 (s)	6.55 (s)	6.27 (s)	6.30 (s)
23	7.53 (s)	7.41 (s)	7.40 (s)		
28	0.92 (s)	0.84 (s)	0.72 (s)	0.90 (s)	0.90 (s)
29a	1.89 (d, 10.8)	1.95 (d, 11.2)	1.97 (m)	1.90 (d, 10.9)	1.90 (d, 10.8)
29b	1.77 (d, 10.8)	1.85 (d, 11.1)	1.71 (d, 11.5)	1.75 (d, 10.9)	1.76 (d, 10.8)
30	6.01 (s)	5.30 (s)	5.39 (s)	5.85 (s)	5.90 (s)
32	1.61 (s)	1.51 (s)	1.69 (s)	1.66 (s)	1.67 (s)
MeO-7	3.69 (s)	3.64 (s)	3.67 (s)	3.72 (s)	3.73 (s)
R-3					
2′	2.83 (m)	2.98 (m)	2.48 (m)	2.88 (m)	2.52 (m)
3′	1.19 (d, 6.5)	1.29 (d, 6.8)	1.19 (d, 6.6)	1.09 (d, 7.5)	1.09 (d, 7.2)
4′	1.26 (d, 6.5)	1.24 (d, 6.8)	1.13 (d, 6.6)	1.11 (d, 7.5)	1.18 (d, 7.2)
R-2/30					
2″	2.73 (m)	2.62 (m)	2.43 (m)	2.34 (m)	2.92 (m)
3″	1.14 (d, 6.9)	1.20 (d, 6.8)	1.16 (t, 7.6)	1.06 (t, 7.5)	1.09 (d, 6.7)
4″	1.20 (d, 6.9)	1.21 (d, 6.8)			1.12 (d, 6.7)
R-15					
2‴	2.63 (m)				
3‴	1.25 (d, 7.0)				
4‴	1.22 (d, 7.0)				
OAc-12			1.52 (s)		

**Table 2:** ^13^C NMR (125 MHz) data of compounds **1**–**5** (CDCl_3_, *δ*_C_ in ppm).

Carbon	1	2	3	4	5
1	85.5	84.7	84.4	85.5	85.5
2	80.3	74.8	83.5	79.4	79.7
3	83.3	86.4	84.7	83.1	82.7
4	45.2	43.9	44.9	45.4	45.5
5	37.3	38.3	40.5	37.2	37.3
6	33.6	33.7	33.2	33.9	33.9
7	173.6	173.7	174.3	175.2	175.1
8	85.3	83.6	83.8	85.9	86.1
9	86.2	82.5	86.1	86.8	86.9
10	45.8	45.7	48.1	45.4	45.4
11	34.7	26.8	32.8	25.4	25.4
12	66.5	71.7	68.9	28.8	28.8
13	42.4	43.0	43.1	34.7	34.8
14	52.8	47.2	152.6	42.7	42.6
15	64.0	30.5	124.0	26.6	26.5
16	168.4	171.6	163.5	169.2	169.2
17	76.6	76.5	78.9	78.3	78.1
18	12.8	22.6	14.6	20.3	20.2
19	16.5	14.6	15.6	16.5	16.5
20	121.3	115.7	121.3	163.6	163.6
21	141.6	141.1	142.2	97.5	97.5
22	109.3	110.6	110.4	122.2	122.3
23	144.5	143.4	143.1	169.1	169.0
28	15.0	14.8	14.4	14.5	14.5
29	39.6	40.5	40.0	39.7	39.7
30	70.8	65.5	74.4	70.6	70.4
31	119.6	119.9	119.9	119.2	119.3
32	21.1	21.4	16.7	21.2	21.2
MeO-7	52.4	52.4	52.3	53.0	53.0
R-3					
1′	176.6	176.6	176.1	177.3	177.1
2′	34.6	34.2	34.0	34.1	34.7
3′	18.4	18.9	18.5	19.9	20.0
4′	19.9	19.8	20.4	18.1	18.1
R-2/30					
1″	175.8	175.7	173.7	172.6	175.2
2″	34.7	34.1	28.4	27.9	34.0
3″	18.3	19.1	9.16	8.7	18.1
4″	19.3	19.3			19.5
R-15					
1‴	175.1				
2‴	33.9				
3‴	19.0				
4‴	19.1				
OAc-12			170.6, 20.1		

**Table 3:** In vitro anti-inflammatory activity of compounds **1**–**5**.

Compound	IC_50_ Value (μM) ^a^
**1**	21.72 ± 2.79
**2**	23.29 ± 1.01
**3**	47.08 ± 3.47
**4**	–
**5**	66.67 ± 2.89
Quercetin ^b^	8.28 ± 0.81

^a^ Values present mean ± SD of triplicate experiments. ^b^ Positive control; “–” inactive.

## Data Availability

The datasets generated and analyzed during the current study are available from the corresponding author on reasonable request.

## Supplementary Materials

The following are available online at https://www.mdpi.com/article/10.3390/molecules28135136/s1, Figures S1–S35: ^1^H, ^13^C, HSQC, ^1^H-^1^H COSY, HMBC, ROESY and HRESIMS spectra of the new compounds **1**–**6.**

## Sample Availability

Available.
