# Supplementary material for: Phragmalin-Type Limonoids from the Fruits of Chukrasia tabularis and Their Anti-Inflammatory Activity
Source: Molecules. 2023 Jun 30;28(13):5136. doi: 10.3390/molecules28135136 (PMC10343657; doi:10.3390/molecules28135136)
Supplement: Supplementary file 1 [file molecules-28-05136-s001.zip › molecules-2434682-supplementary.pdf]

Supporting Information

Phragmalin-type limonoids from the fruits of *Chukrasia tabularis* and their anti-inflammatory activity

Shu-Jun Dai [1], Yu-Zhen Wu [1], Xiu-Juan Xin [1] and Fa-Liang An [1,2*]

[1] State Key Laboratory of Bioreactor Engineering, East China University of Science and Technology, 130 Meilong Road, Shanghai 200237, China

[2] Marine Biomedical Science and Technology Innovation Platform of Lin-gang Special Area, No.4, Lane 218, Haiji Sixth Road, Shanghai 201306, China

*Correspondence: flan2016@ecust.edu.cn; Tel.: +86-21-6425-3823

Contents

Figure S1. ^{1}H NMR (500 MHz, $CDCl_3$) spectrum of compound **1**
Figure S2. ^{13}C NMR (125 MHz, $CDCl_3$) spectrum of compound **1**
Figure S3. HSQC spectrum of compound **1** in $CDCl_3$
Figure S4. ^{1}H-^{1}H COSY spectrum of compound **1** in $CDCl_3$
Figure S5. HMBC spectrum of compound **1** in $CDCl_3$
Figure S6. ROESY spectrum of compound **1** in $CDCl_3$
Figure S7. HRESIMS spectrum of compound **1**
Figure S8. ^{1}H NMR (500 MHz, $CDCl_3$) spectrum of compound **2**
Figure S9. ^{13}C NMR (125 MHz, $CDCl_3$) spectrum of compound **2**
Figure S10. HSQC spectrum of compound **2** in $CDCl_3$
Figure S11. ^{1}H-^{1}H COSY spectrum of compound **2** in $CDCl_3$
Figure S12. HMBC spectrum of compound **2** in $CDCl_3$
Figure S13. ROESY spectrum of compound **2** in $CDCl_3$
Figure S14. HRESIMS spectrum of compound **2**
Figure S15. ^{1}H NMR (500 MHz, $CDCl_3$) spectrum of compound **3**
Figure S16. ^{13}C NMR (125 MHz, $CDCl_3$) spectrum of compound **3**
Figure S17. HSQC spectrum of compound **3** in $CDCl_3$
Figure S18. ^{1}H-^{1}H COSY spectrum of compound **3** in $CDCl_3$
Figure S19. HMBC spectrum of compound **3** in $CDCl_3$
Figure S20. ROESY spectrum of compound **3** in $CDCl_3$
Figure S21. HRESIMS spectrum of compound **3**
Figure S22. ^{1}H NMR (500 MHz, $CDCl_3$) spectrum of compound **4**
Figure S23. ^{13}C NMR (125 MHz, $CDCl_3$) spectrum of compound **4**
Figure S24. HSQC spectrum of compound **4** in $CDCl_3$
Figure S25. ^{1}H-^{1}H COSY spectrum of compound **4** in $CDCl_3$
Figure S26. HMBC spectrum of compound **4** in $CDCl_3$
Figure S27. ROESY spectrum of compound **4** in $CDCl_3$
Figure S28. HRESIMS spectrum of compound **4**
Figure S29. ^{1}H NMR (500 MHz, $CDCl_3$) spectrum of compound **5**
Figure S30. ^{13}C NMR (125 MHz, $CDCl_3$) spectrum of compound **5**
Figure S31. HSQC spectrum of compound **5** in $CDCl_3$
Figure S32. ^{1}H-^{1}H COSY spectrum of compound **5** in $CDCl_3$
Figure S33. HMBC spectrum of compound **5** in $CDCl_3$
Figure S34. ROESY spectrum of compound **5** in $CDCl_3$
Figure S35. HRESIMS spectrum of compound **5**

Figure S1. ¹H NMR (500 MHz, $CDCl_3$) spectrum of compound **1**

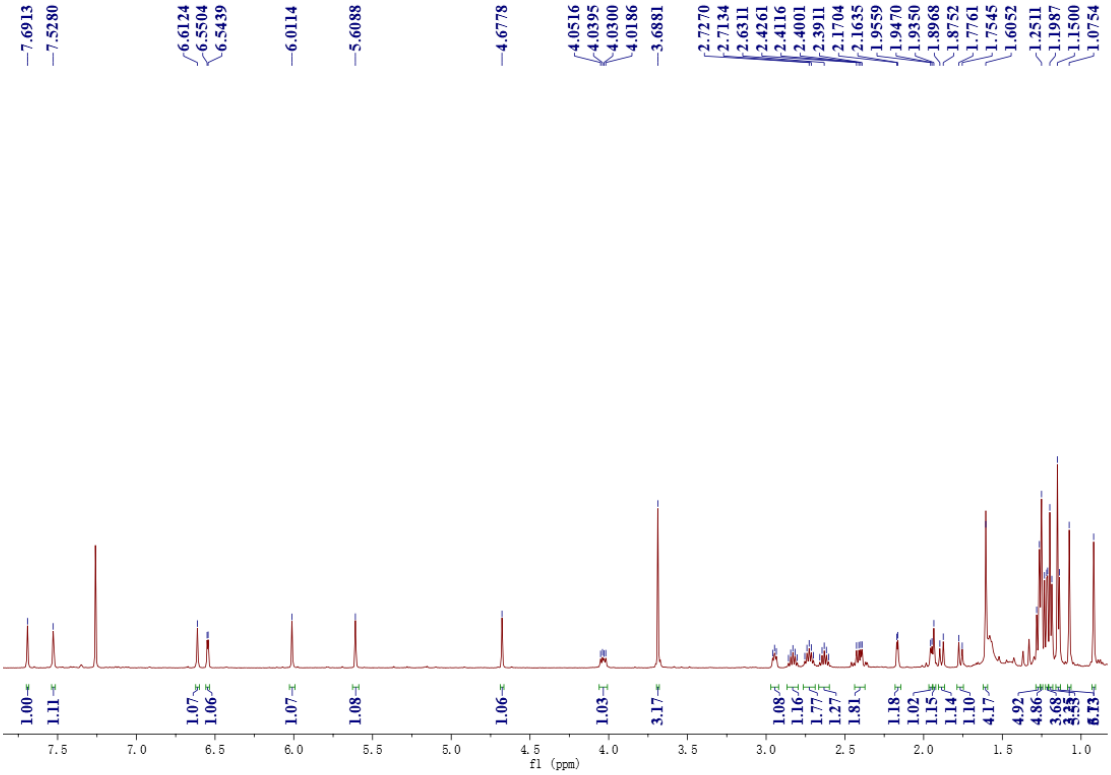

Figure S2. ¹³C NMR (125 MHz, $CDCl_3$) spectrum of compound **1**

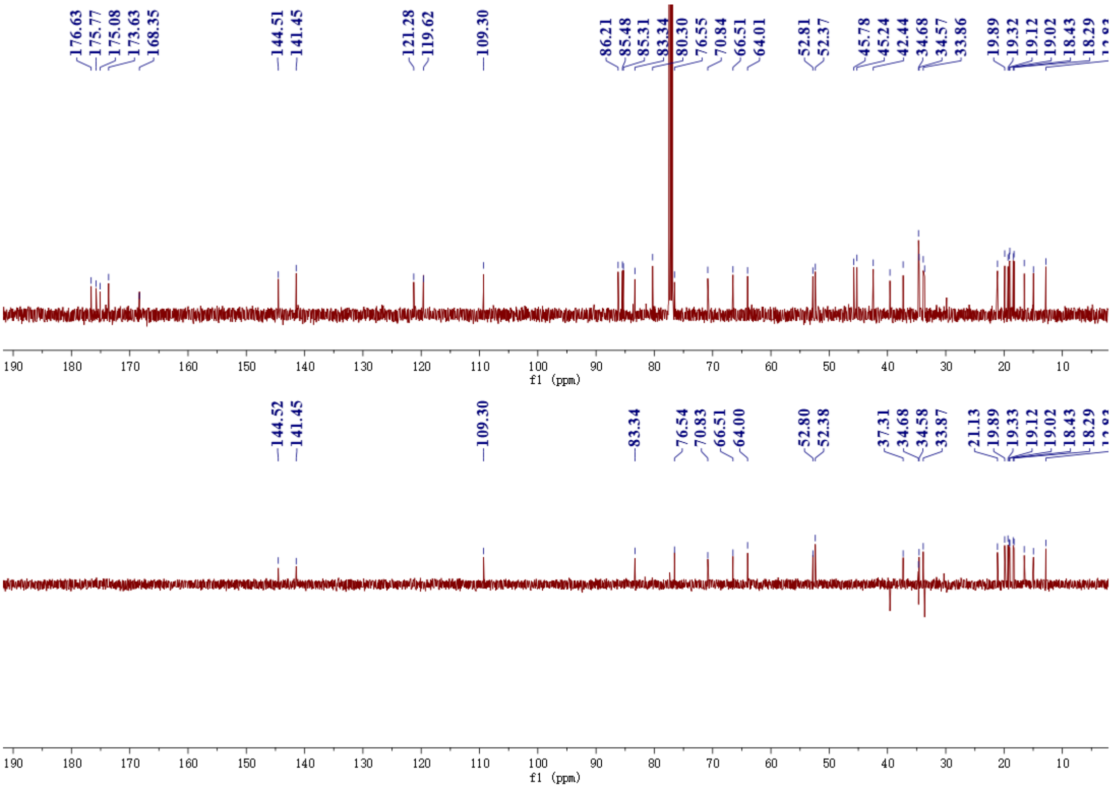

Figure S3. HSQC spectrum of compound **1** in $CDCl_3$

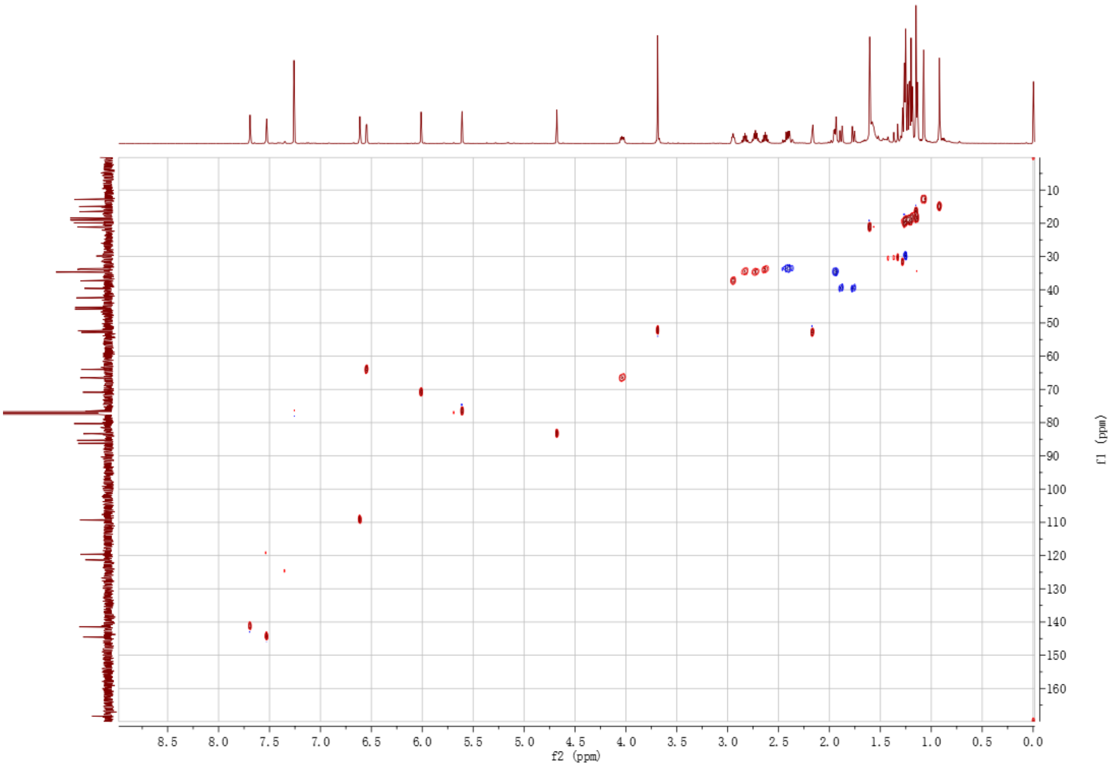

Figure S4. ^1H-^1H COSY spectrum of compound **1** in $CDCl_3$

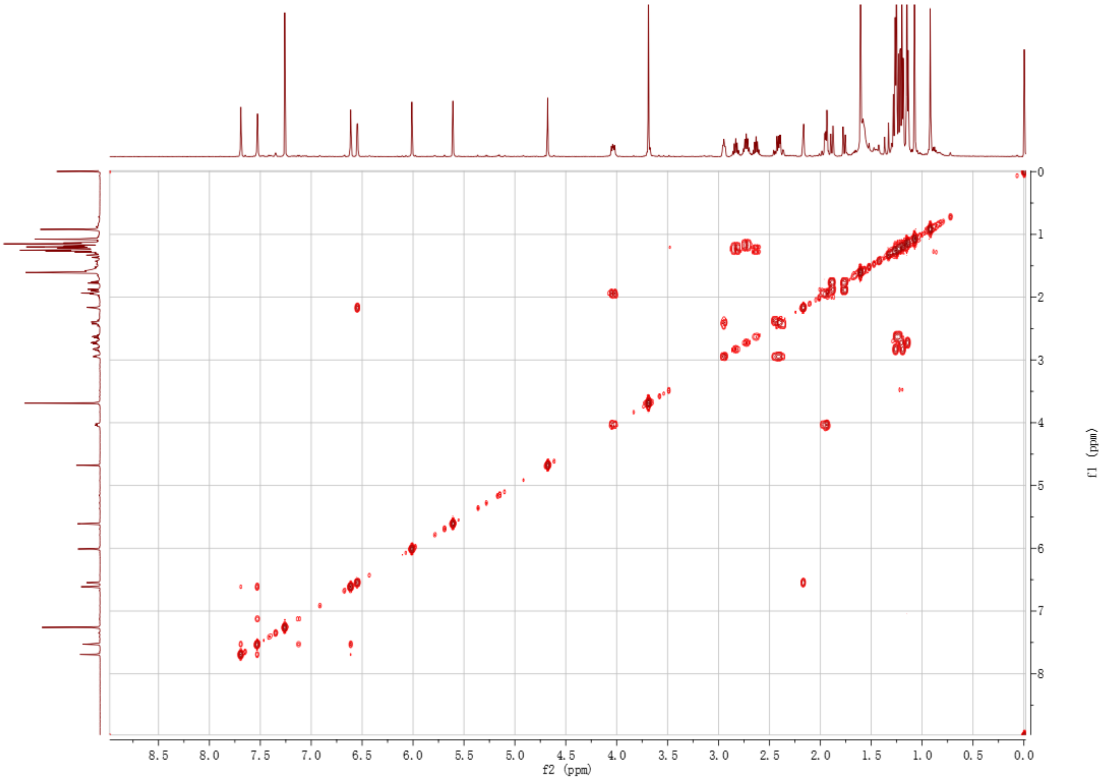

Figure S5. HMBC spectrum of compound **1** in $CDCl_3$

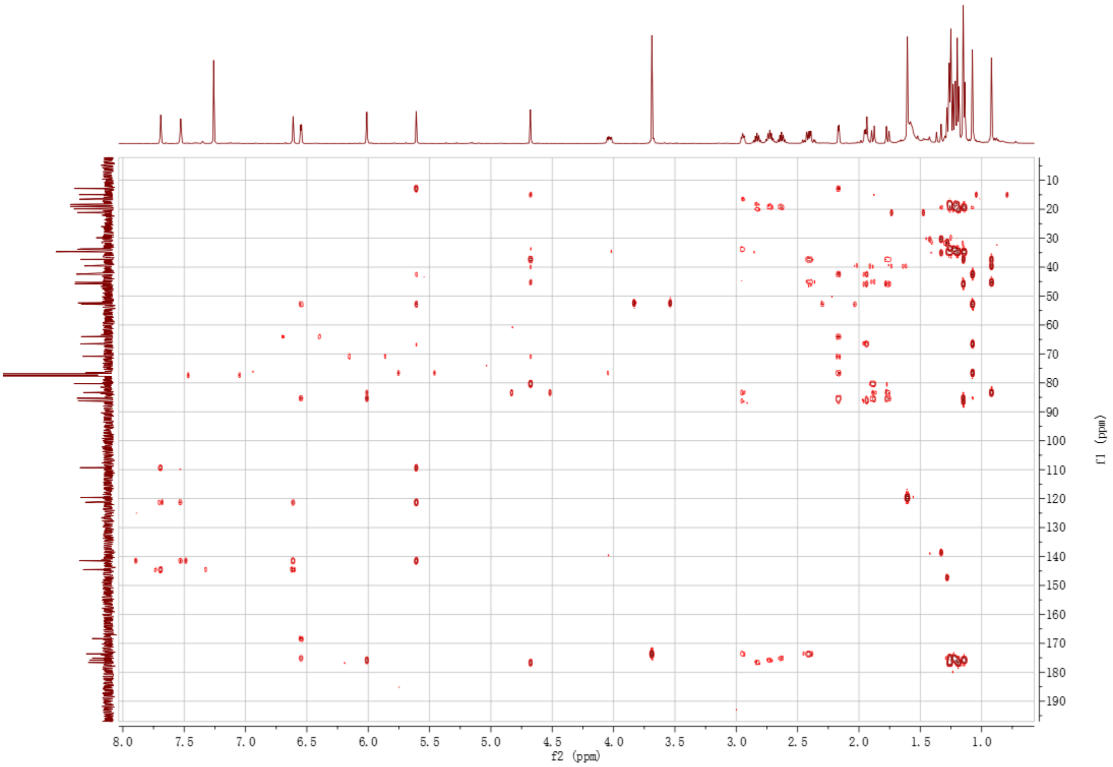

Figure S6. ROESY spectrum of compound **1** in $CDCl_3$

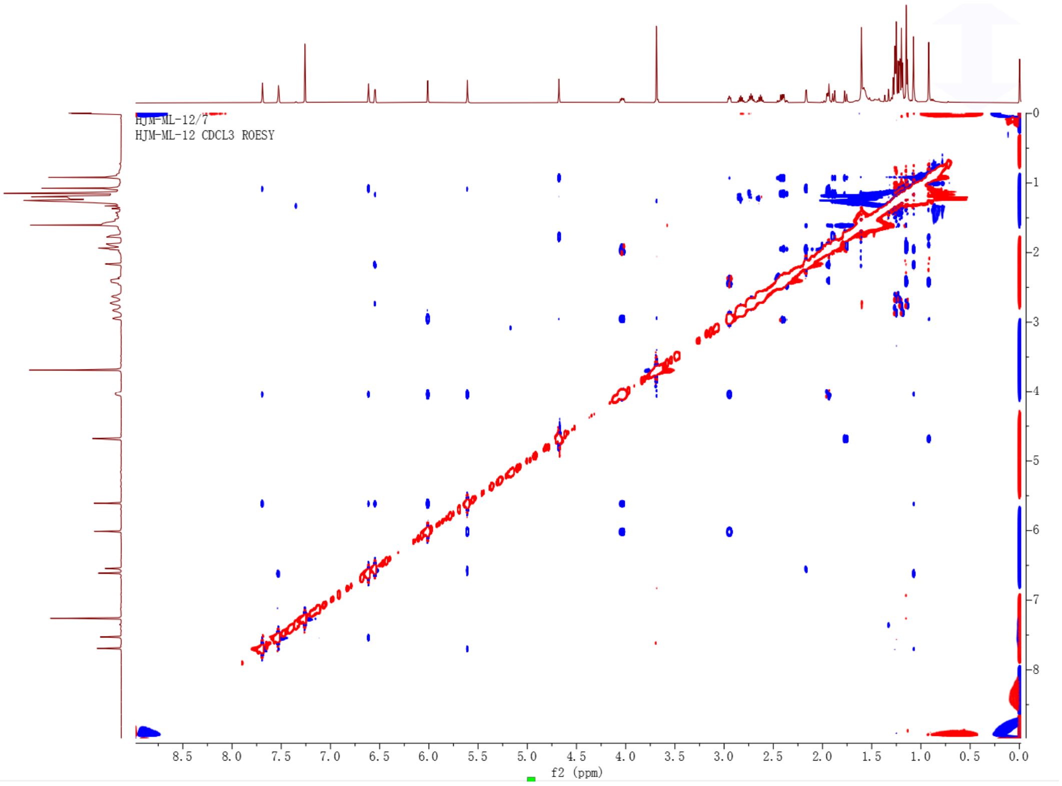

Figure S7. HRESIMS spectrum of compound **1**

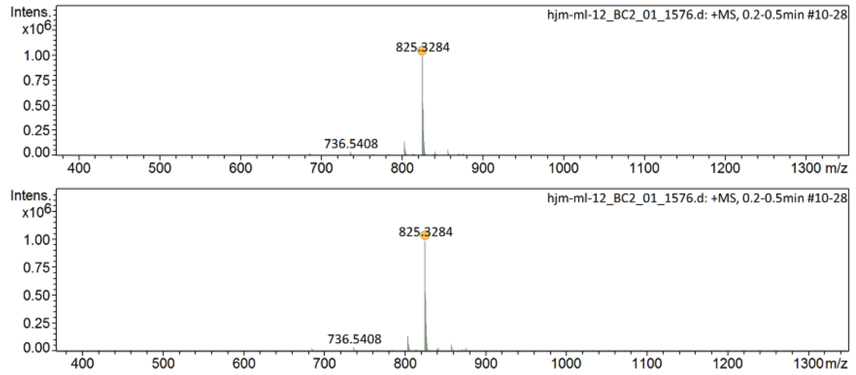

Meas. m/z	#	Ion Formula	m/z	err [ppm]	mSigma	# mSigma	Score	rdb	e¯ Conf	N-Rule	Adduct
825.3284	1	C41H54NaO16	825.3304	2.5	7.6	1	100.00	15.0	even	ok	M+Na

Figure S8. ^{1}H NMR (500 MHz, $CDCl_3$) spectrum of compound **2**

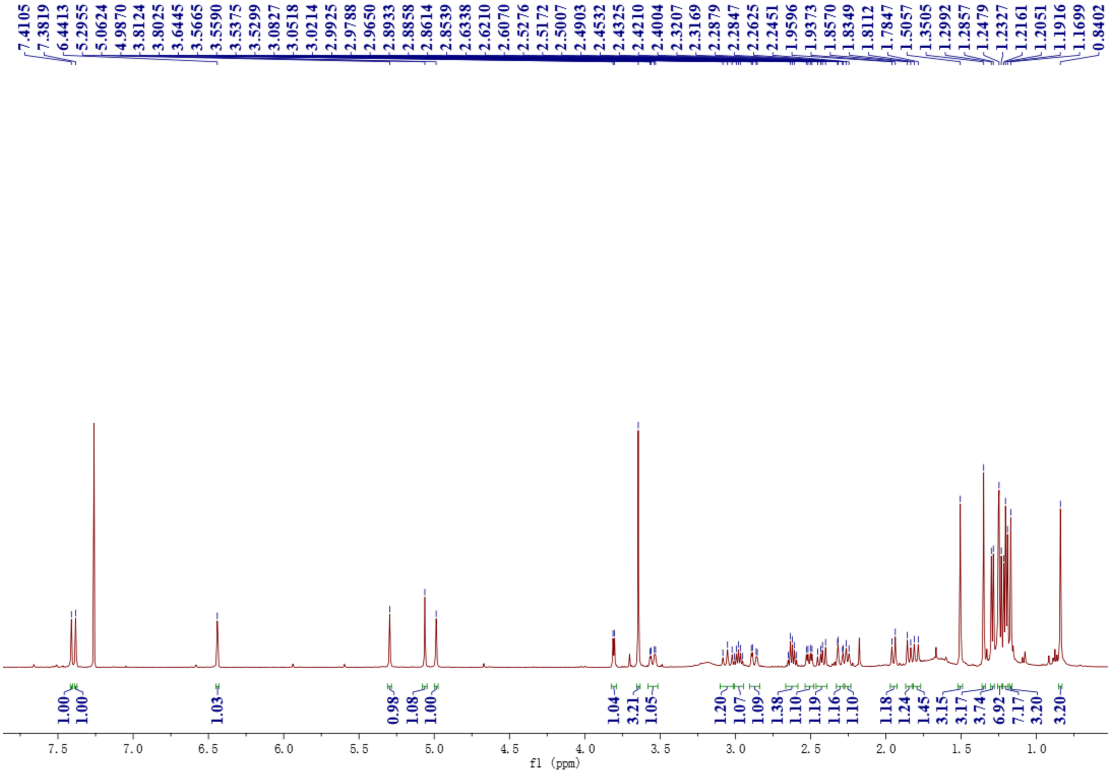

Figure S9. ^{13}C NMR (125 MHz, $CDCl_3$) spectrum of compound **2**

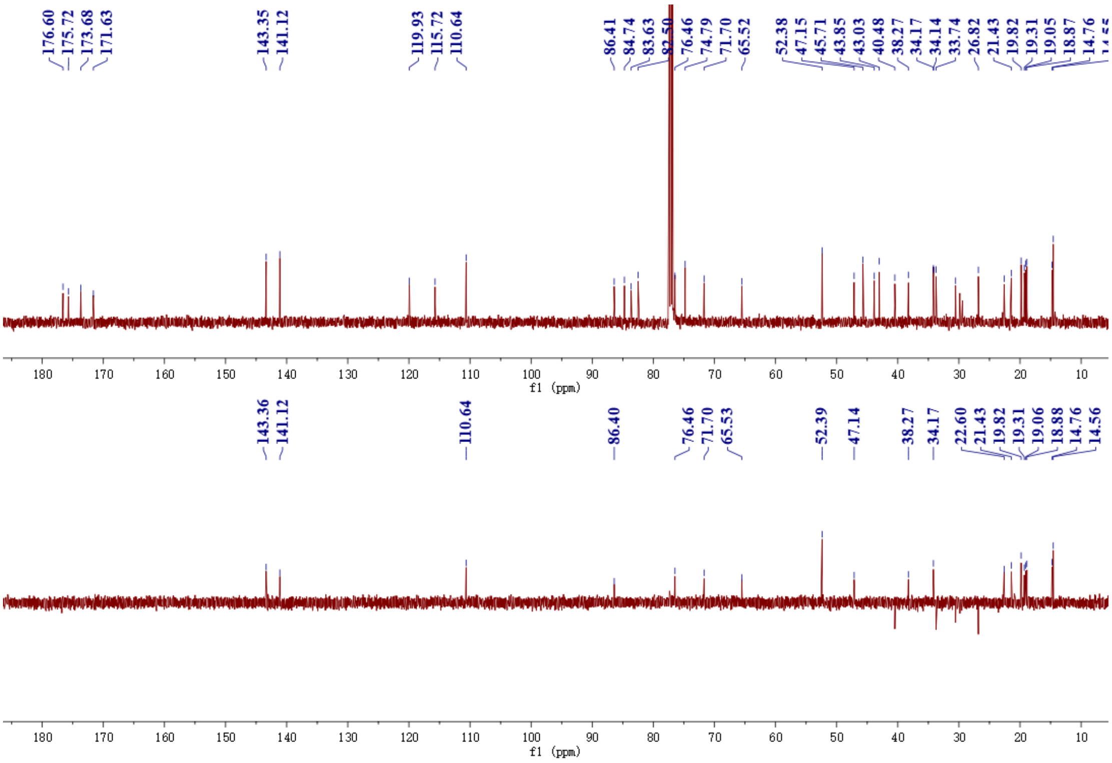

Figure S10. HSQC spectrum of compound **2** in $CDCl_3$

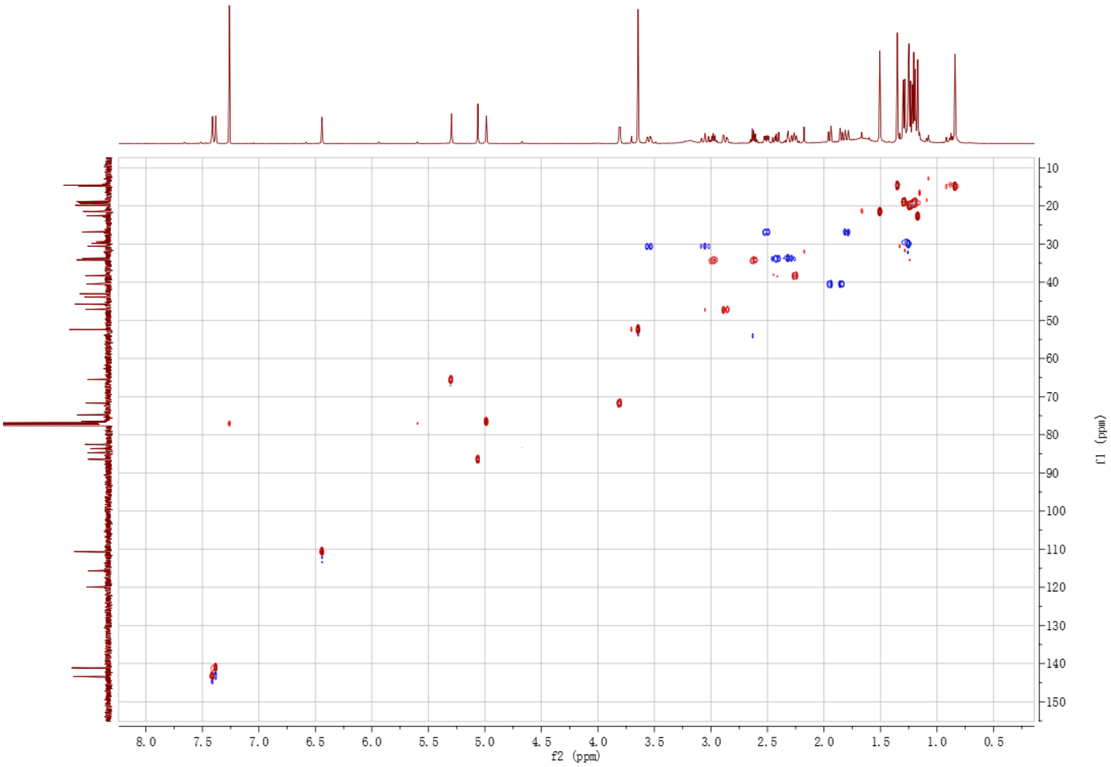

Figure S11. ^{1}H-^{1}H COSY spectrum of compound **2** in $CDCl_3$

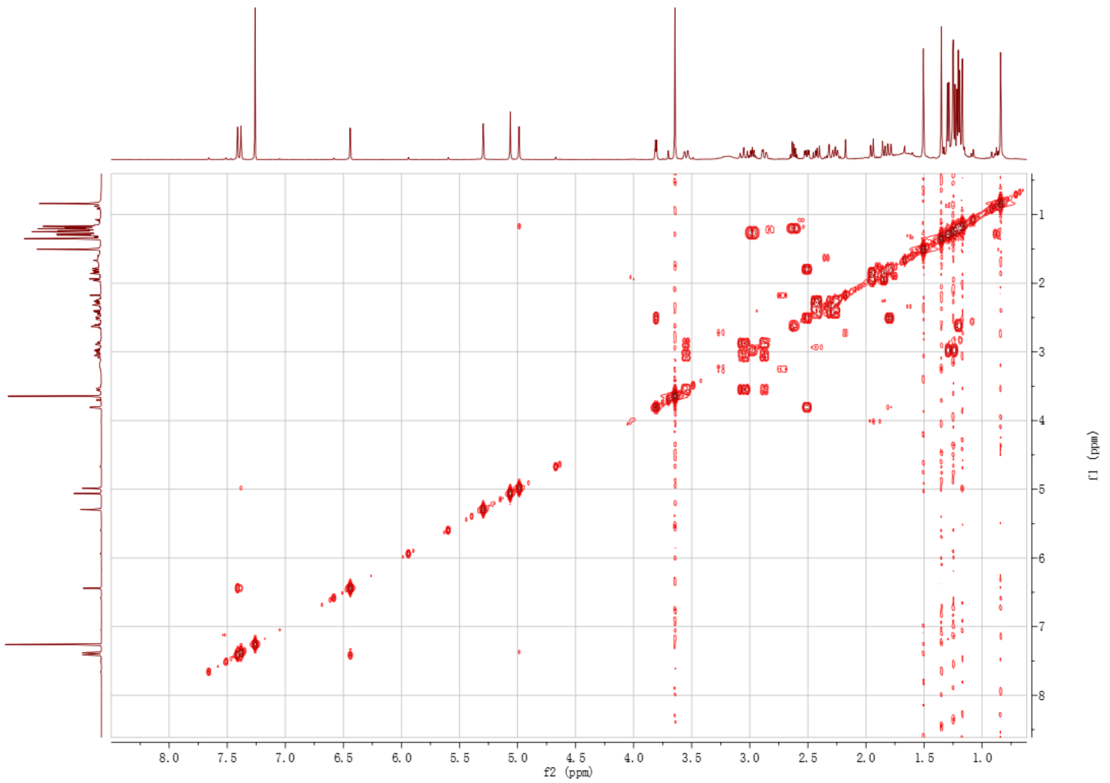

Figure S12. HMBC spectrum of compound **2** in $CDCl_3$

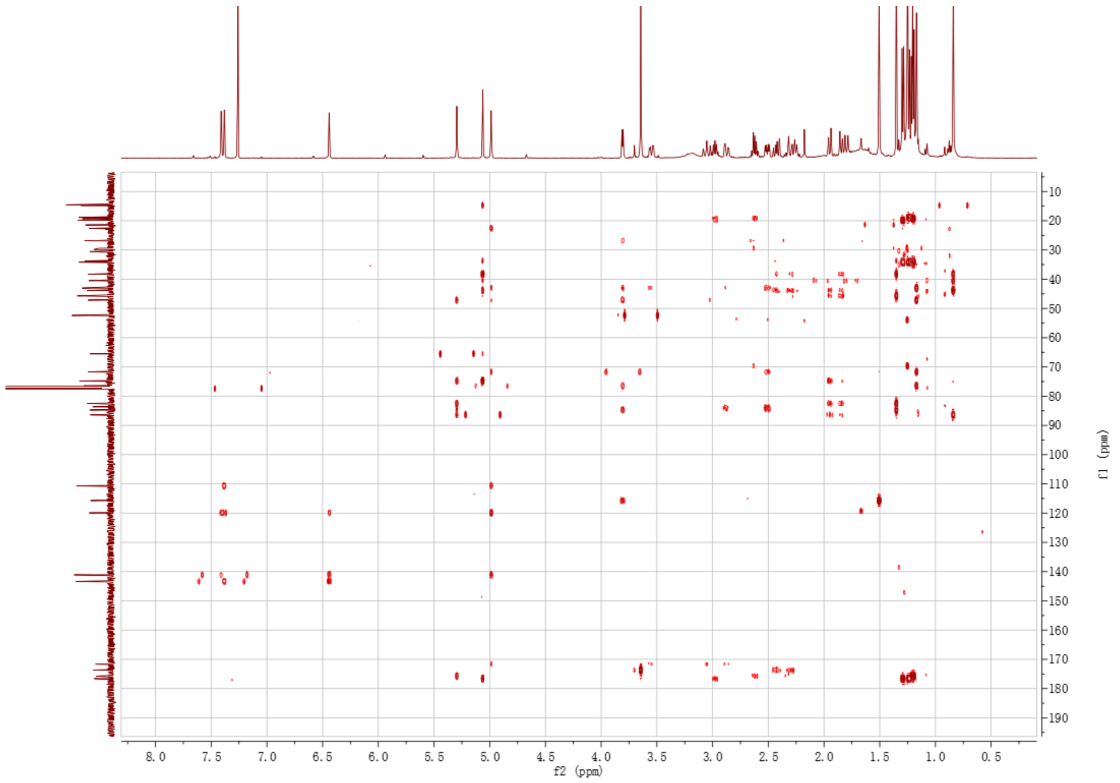

Figure S13. ROESY spectrum of compound **2** in $CDCl_3$

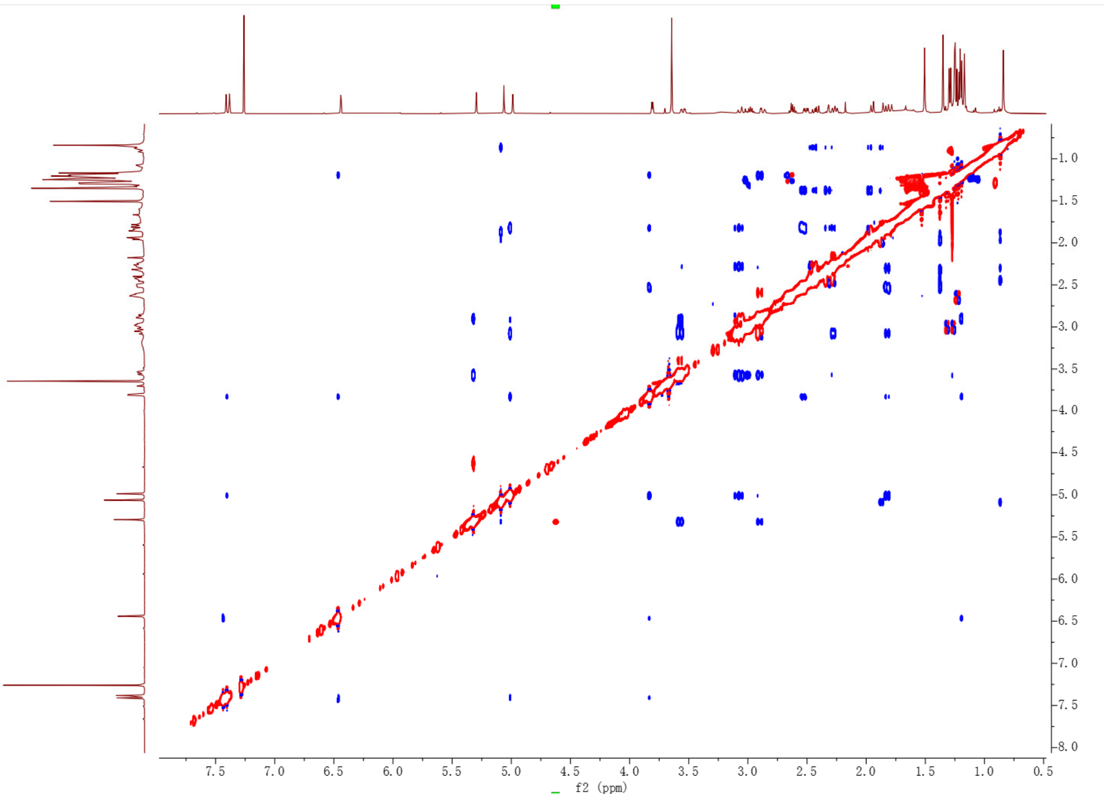

Figure S14. HRESI-MS spectrum of compound **2**

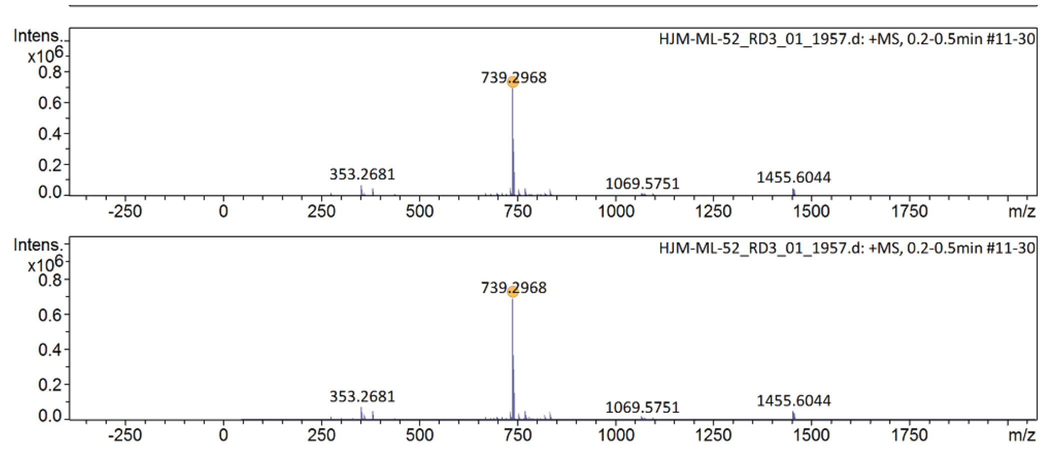

Meas. m/z	#	Ion Formula	m/z	err [ppm]	mSigma	# mSigma	Score	rdb	e⁻ Conf	N-Rule	Adduct
739.2968	1	C37H48NaO14	739.2936	-4.2	3.1	1	100.00	14.0	even	ok	M+Na

Figure S15. ^{1}H NMR (500 MHz, $CDCl_3$) spectrum of compound **3**

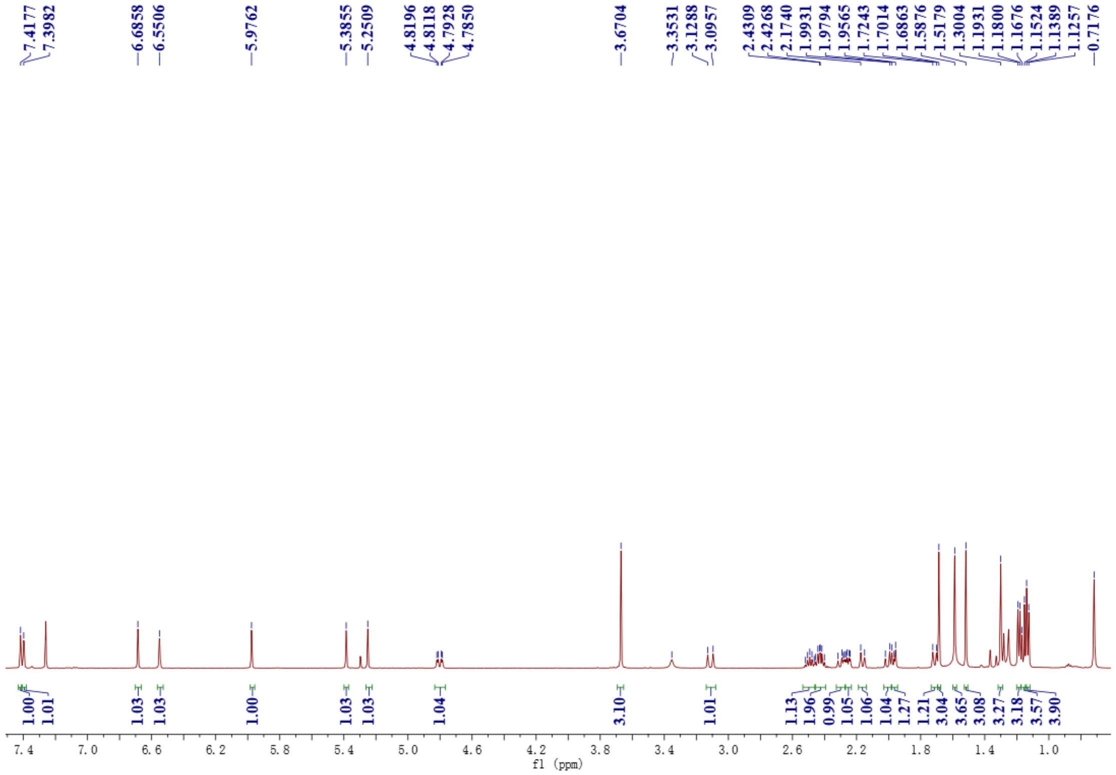

Figure S16. ^{13}C NMR (125 MHz, $CDCl_3$) spectrum of compound **3**

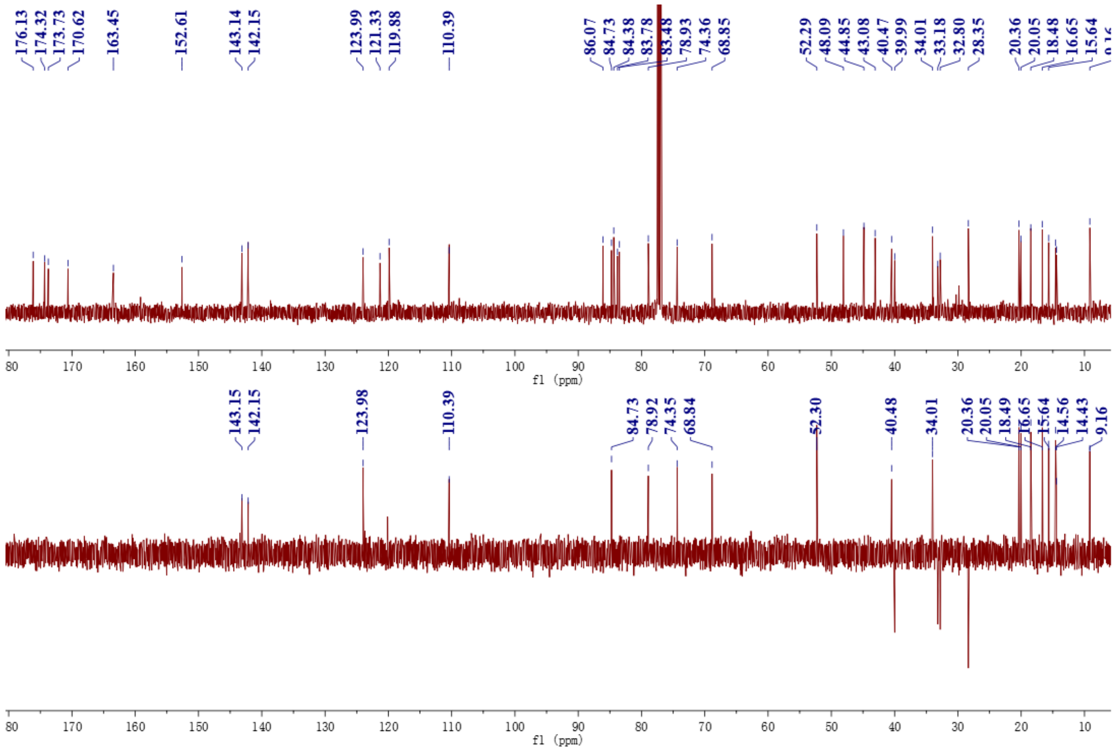

Figure S17. HSQC spectrum of compound **3** in $CDCl_3$

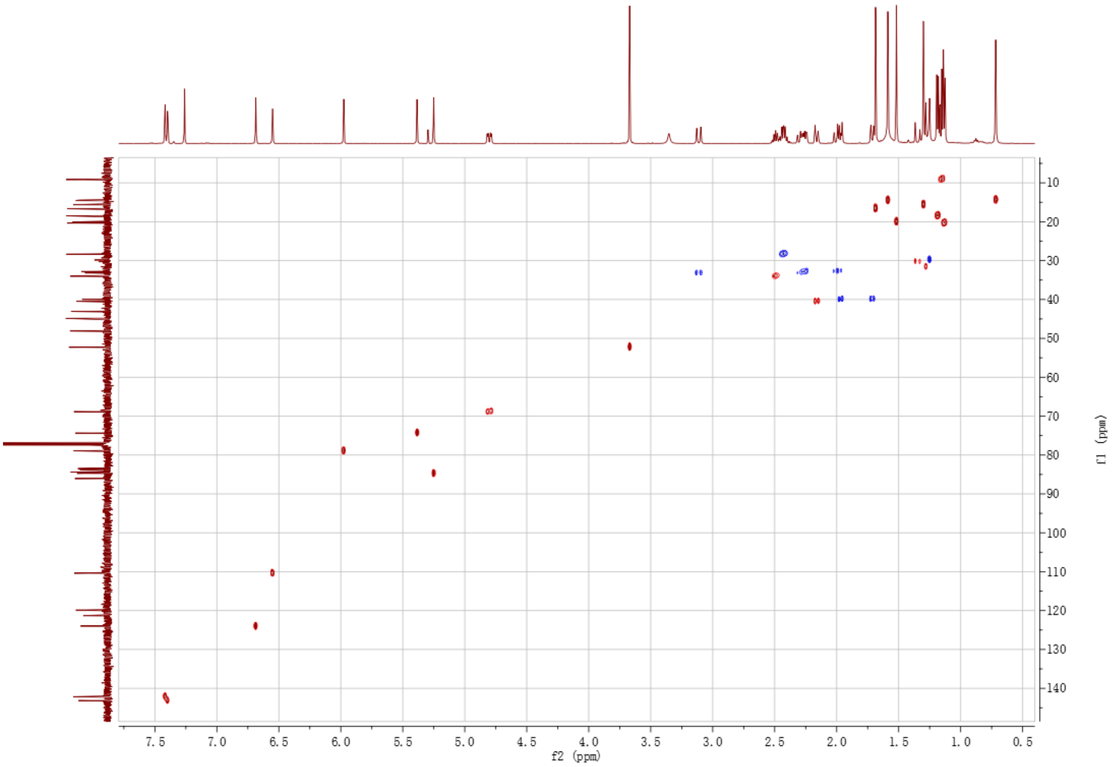

Figure S18. ^1H-^1H COSY spectrum of compound **3** in $CDCl_3$

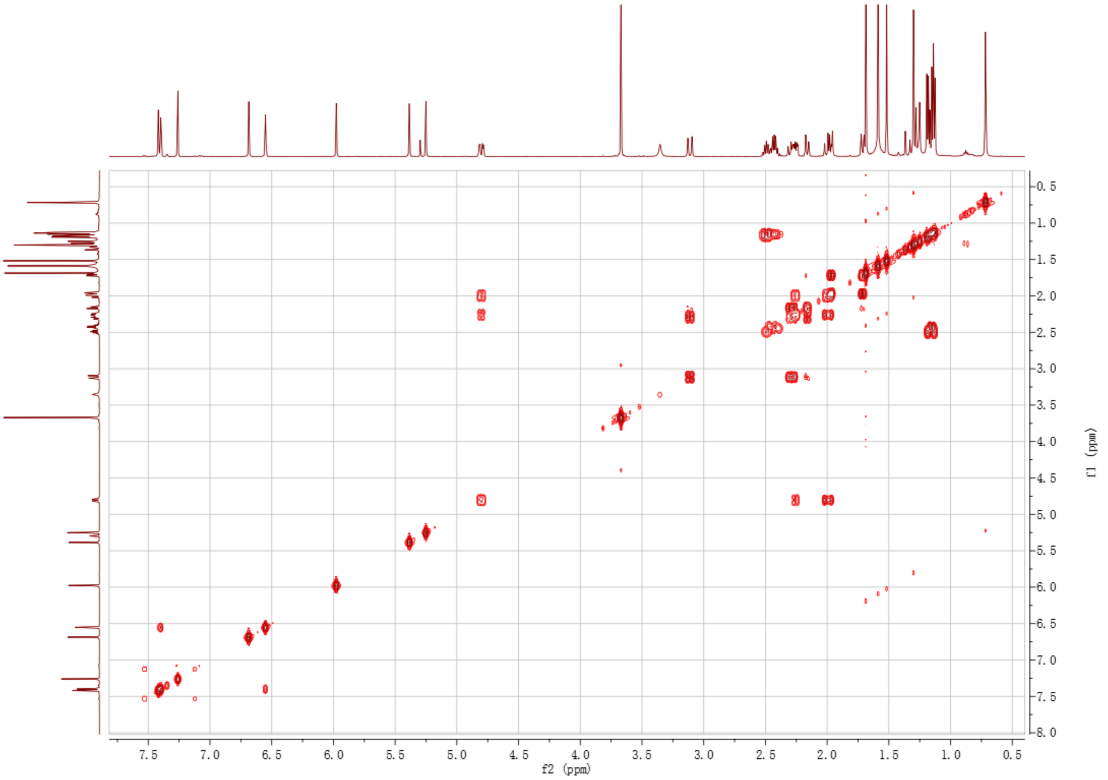

Figure S19. HMBC spectrum of compound **3** in $CDCl_3$

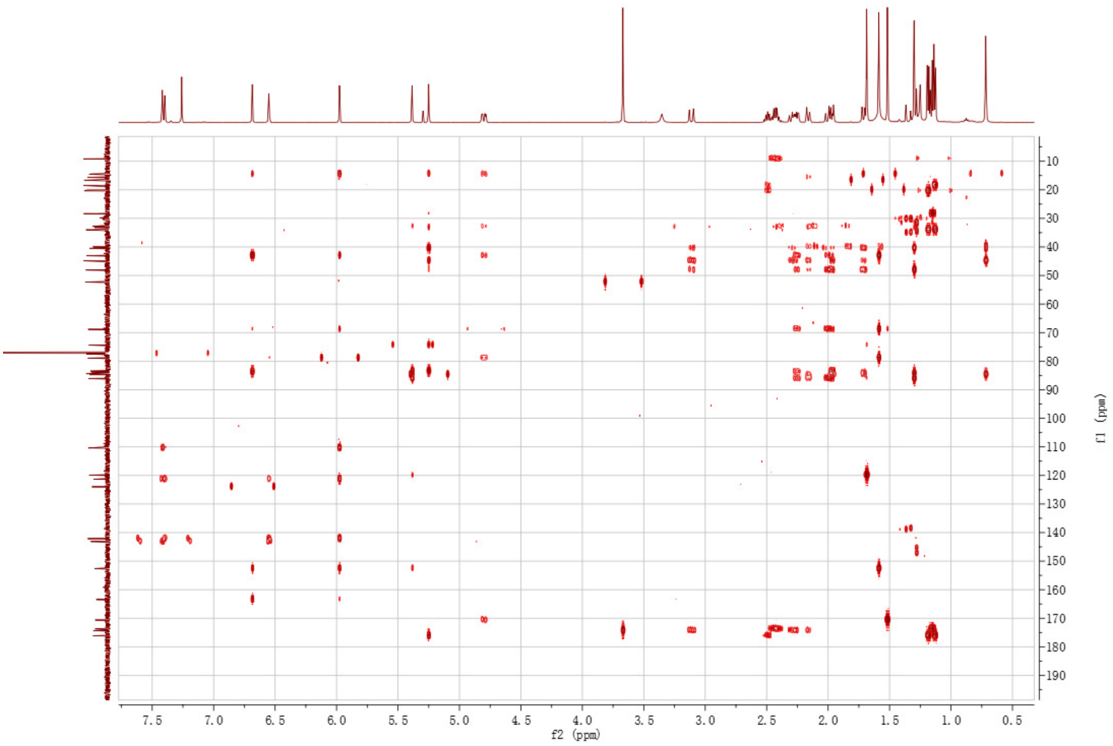

Figure S20. ROESY spectrum of compound **3** in $CDCl_3$

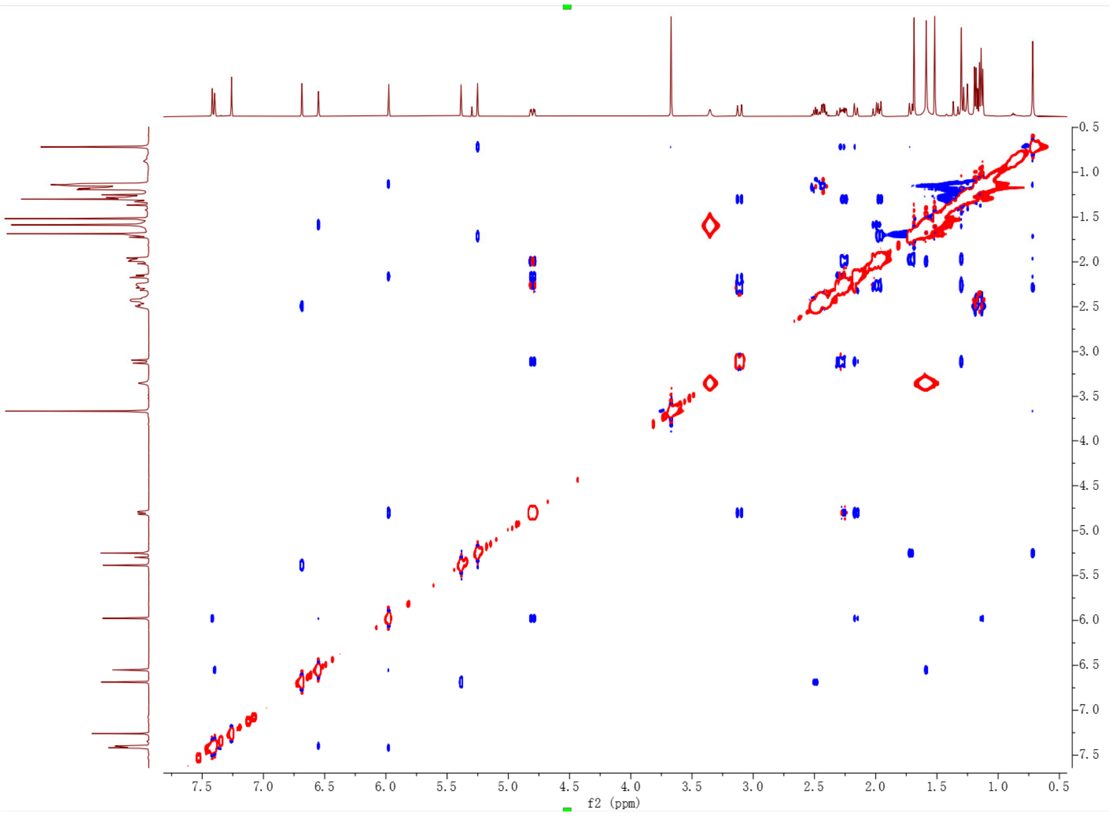

Figure S21. HRESI-MS spectrum of compound **3**

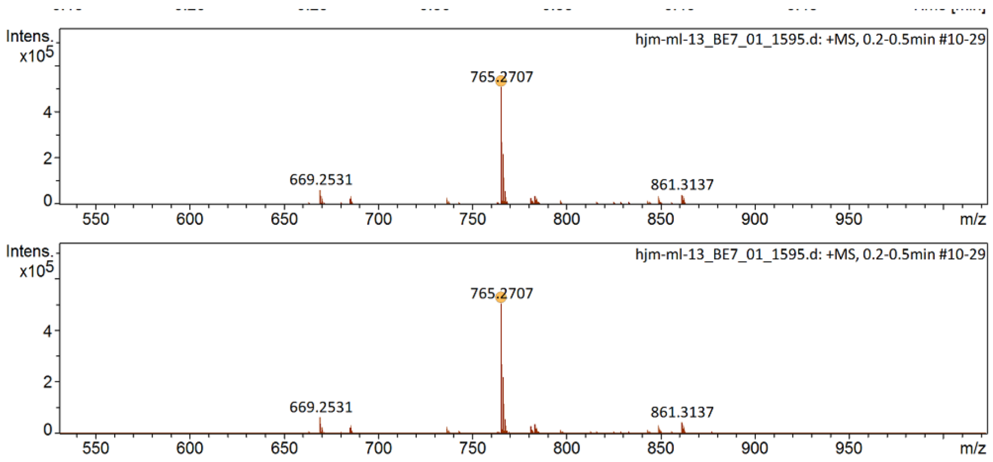

Meas. m/z	#	Ion Formula	m/z	err [ppm]	mSigma	# mSigma	Score	rdb	e¯ Conf	N-Rule	Adduct
765.2707	1	C38H46NaO15	765.2729	2.8	4.8	1	100.00	16.0	even	ok	M+Na

Figure S22. ^{1}H NMR (500 MHz, $CDCl_3$) spectrum of compound **4**

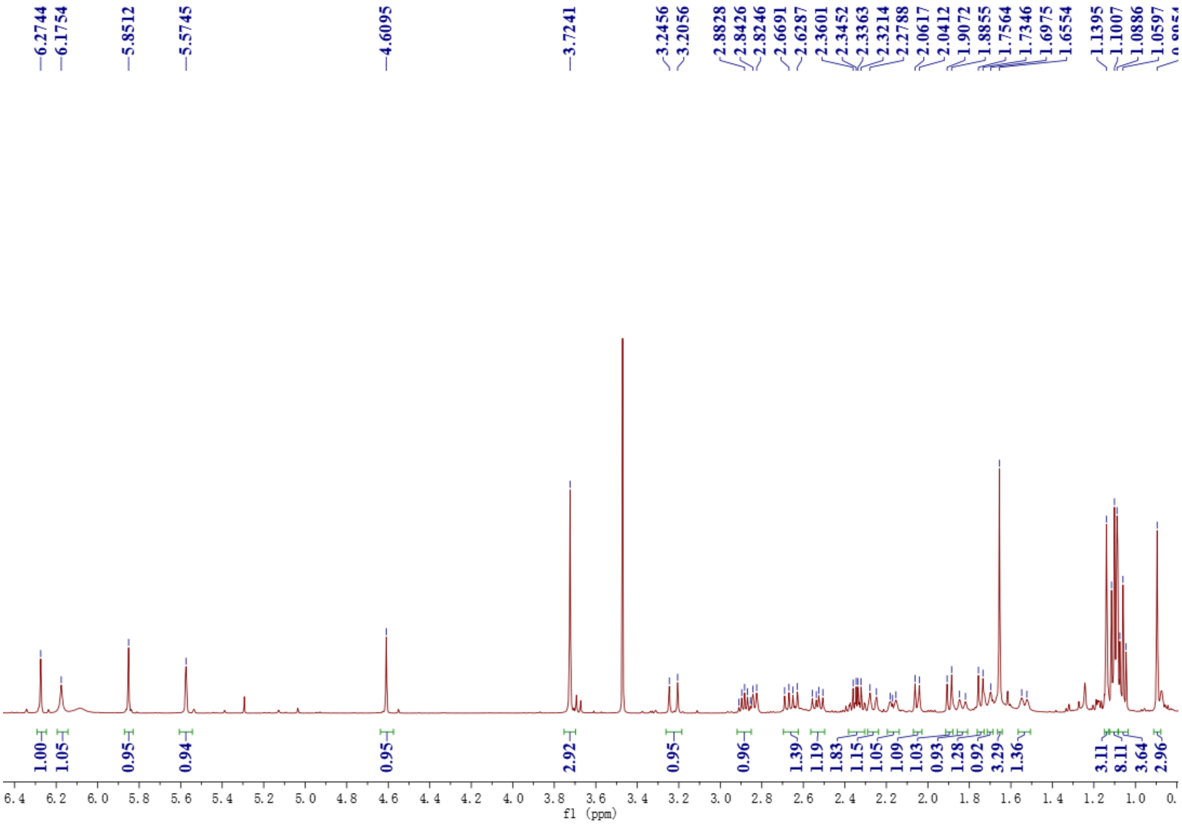

Figure S23. ^{13}C NMR (125 MHz, $CDCl_3$) spectrum of compound **4**

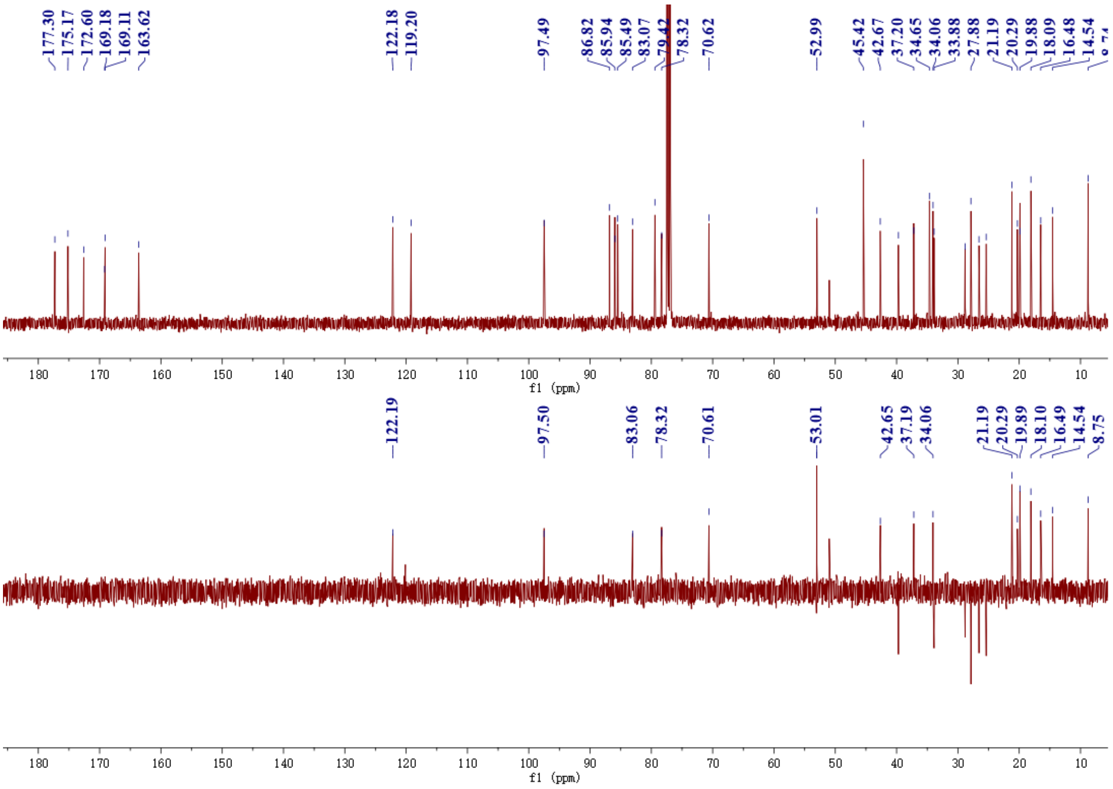

Figure S24. HSQC spectrum of compound **4** in $CDCl_3$

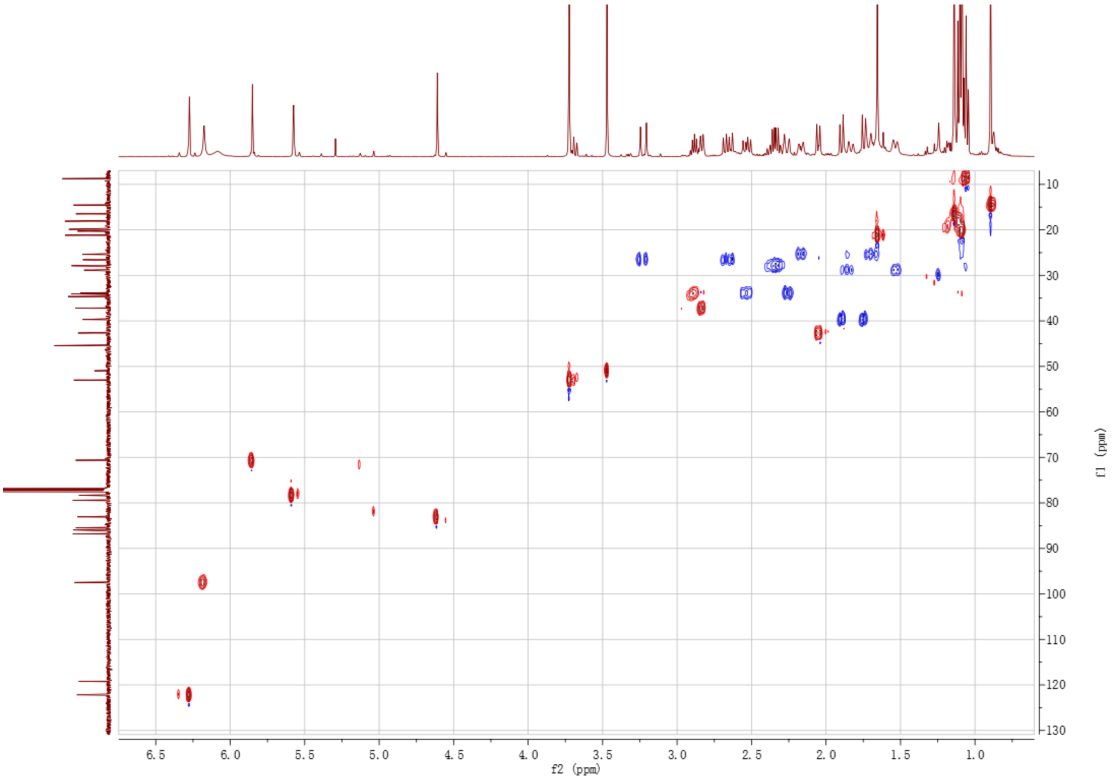

Figure S25. ^{1}H-^{1}H COSY spectrum of compound **4** in $CDCl_3$

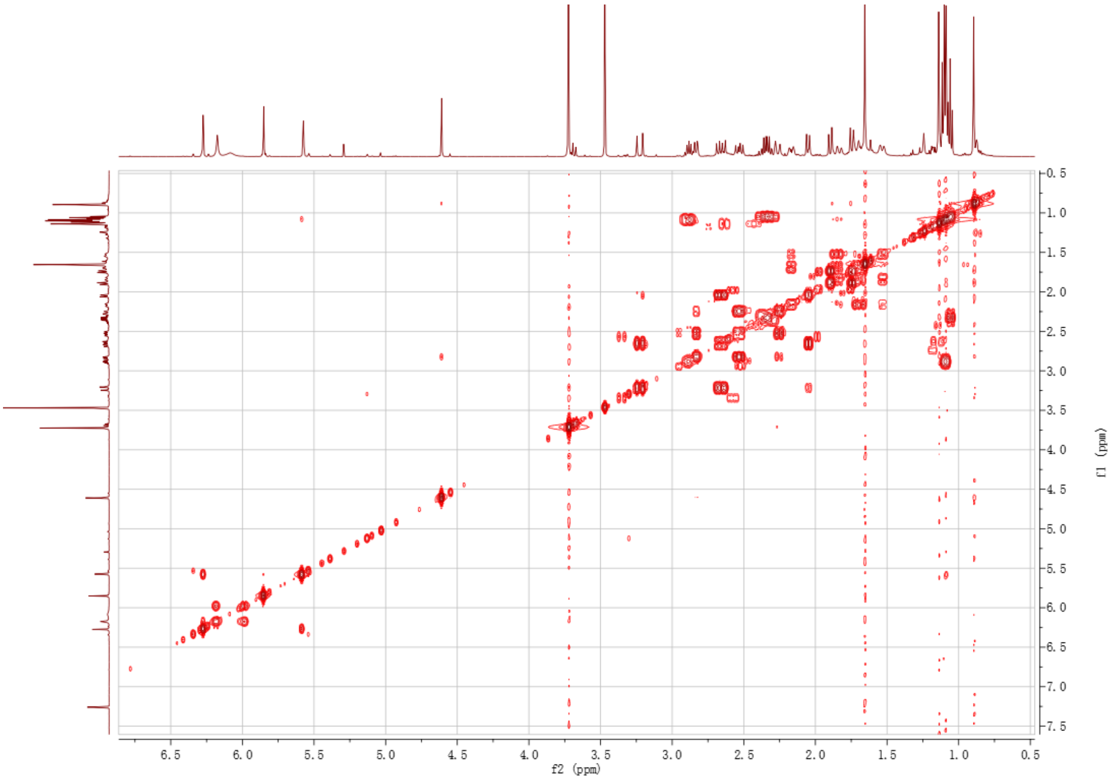

Figure S26. HMBC spectrum of compound **4** in $CDCl_3$

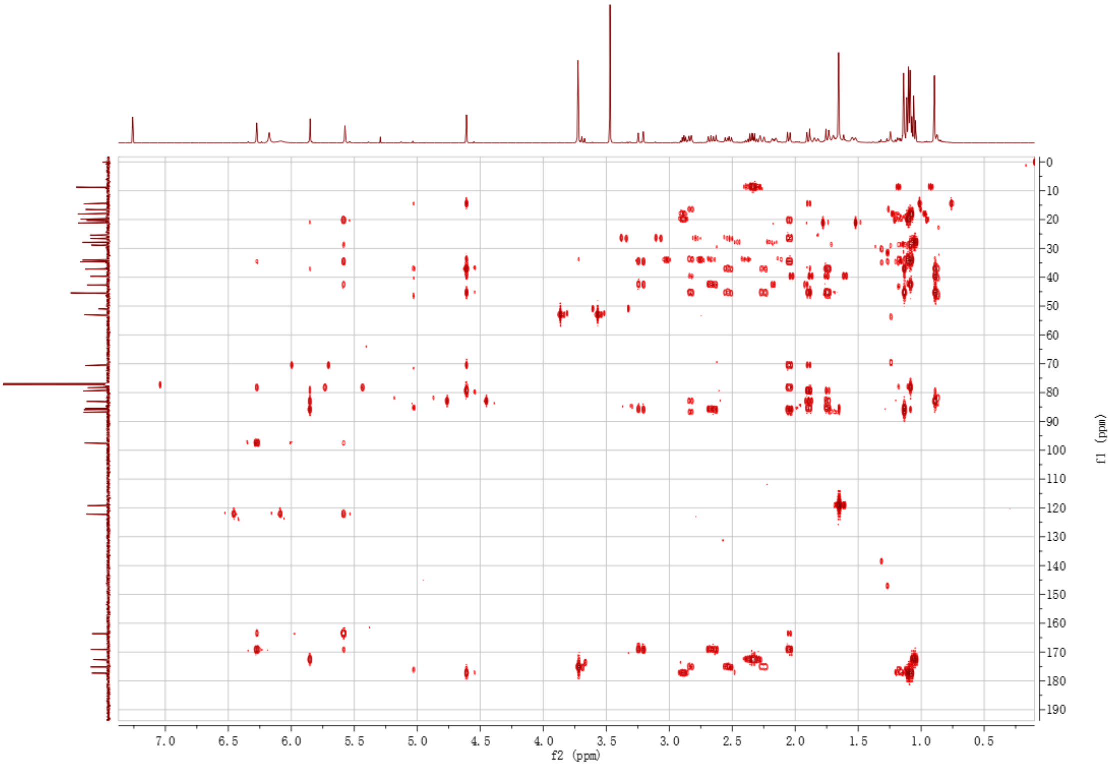

Figure S27. ROESY spectrum of compound **4** in $CDCl_3$

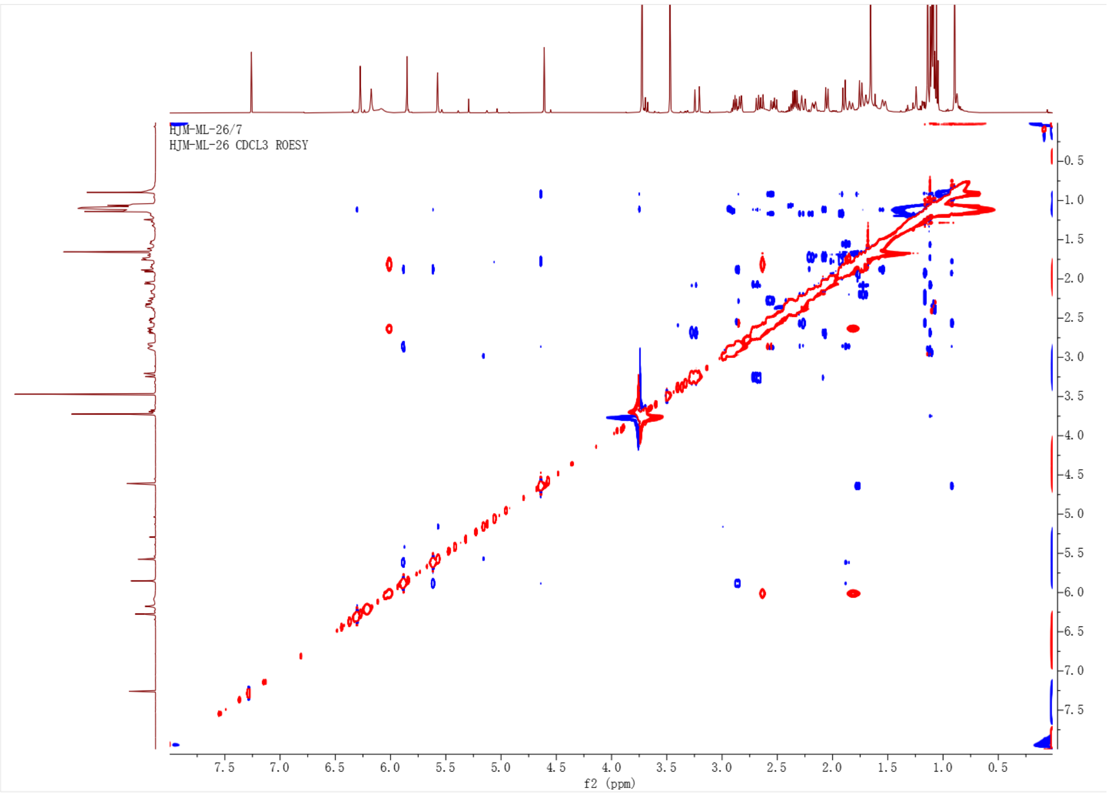

Figure S28. HRESI-MS spectrum of compound **4**

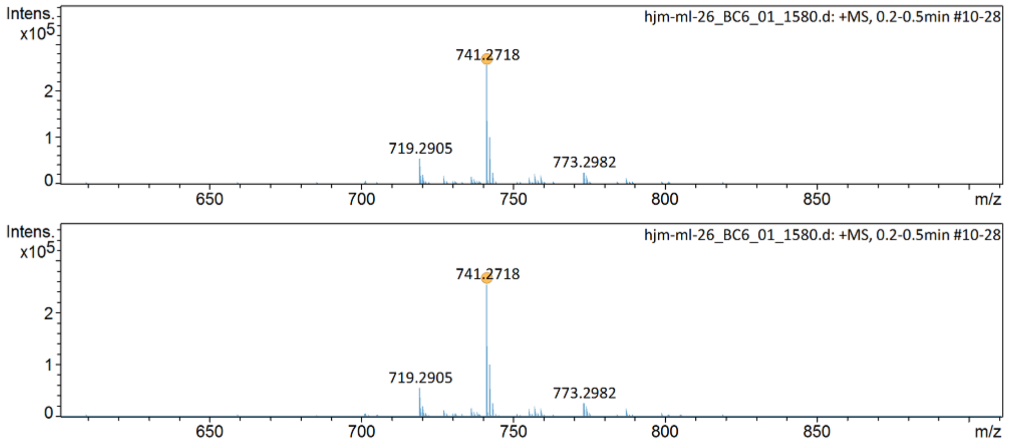

Meas. m/z	#	Ion Formula	m/z	err [ppm]	mSigma	# mSigma	Score	rdb	e⁻ Conf	N-Rule	Adduct
741.2718	1	C36H46NaO15	741.2729	1.5	5.0	1	100.00	14.0	even	ok	M+Na

Figure S29. ^{1}H NMR (500 MHz, $CDCl_3$) spectrum of compound **5**

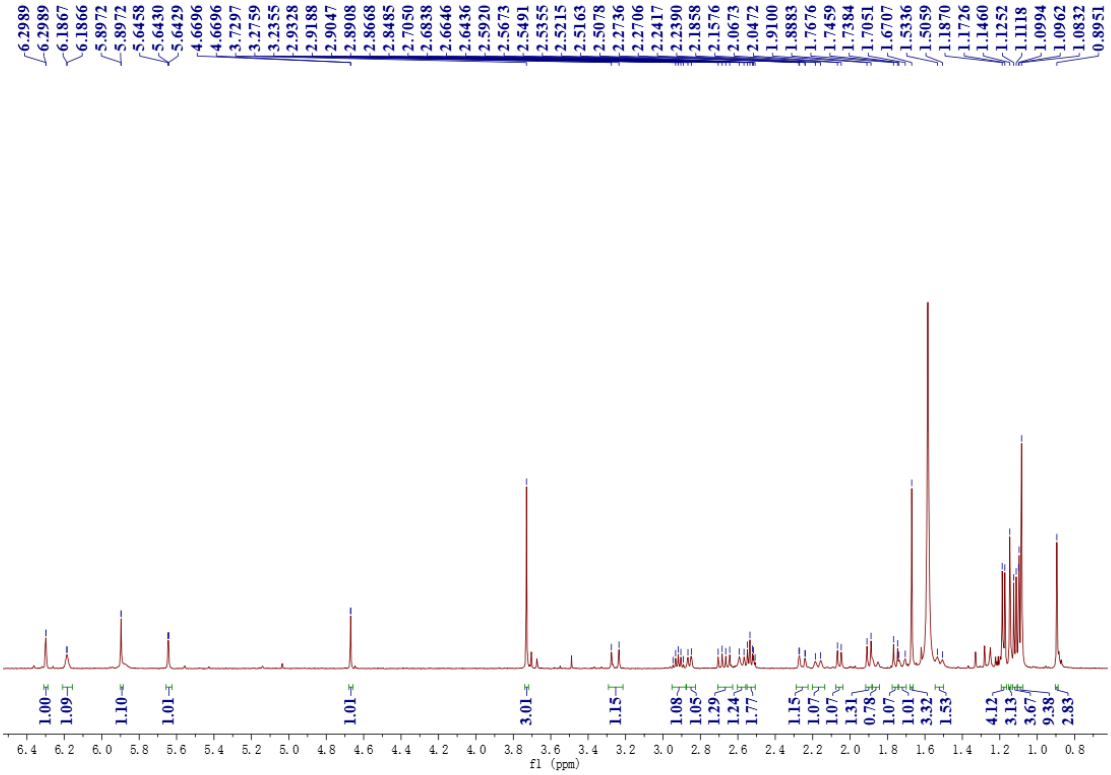

Figure S30. ^{13}C NMR (125 MHz, $CDCl_3$) spectrum of compound **5**

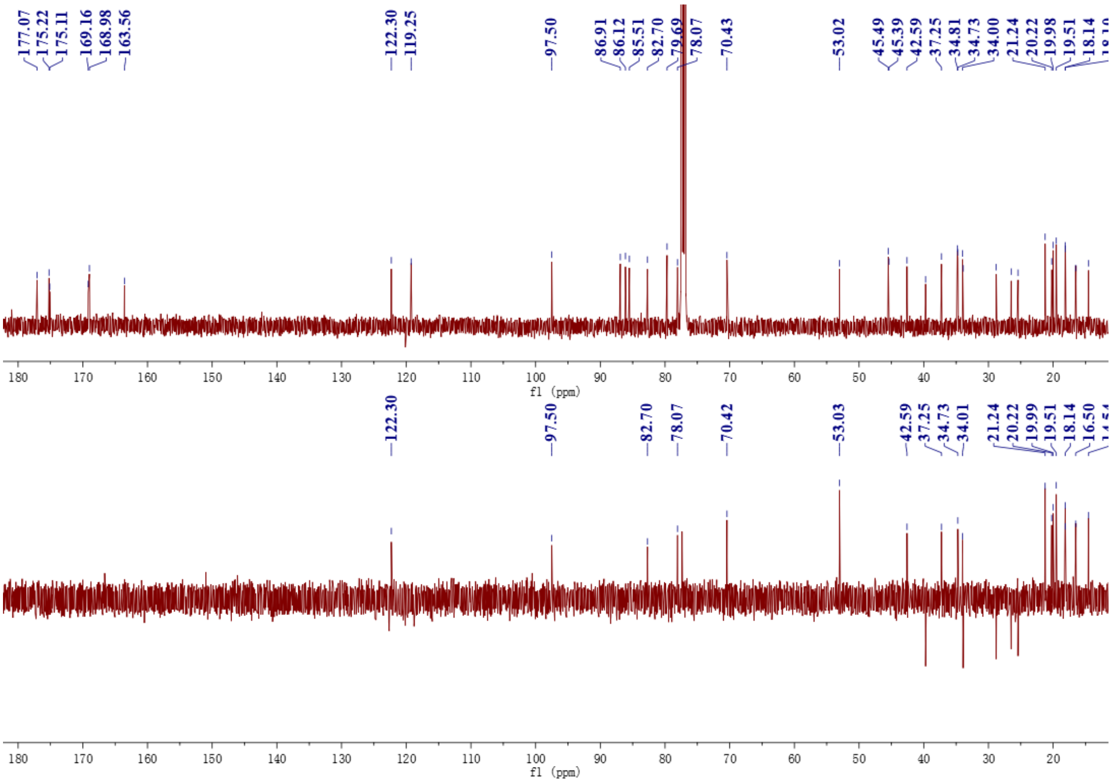

Figure S31. HSQC spectrum of compound **5** in $CDCl_3$

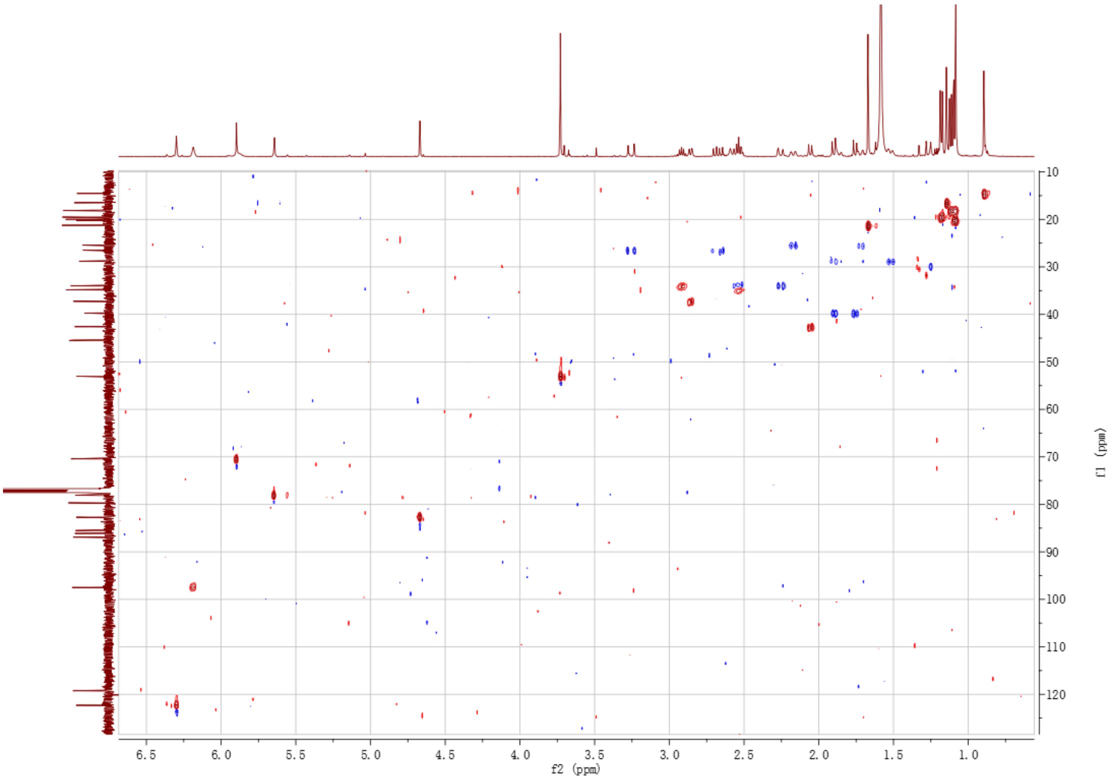

Figure S32. ^1H-^1H COSY spectrum of compound **5** in $CDCl_3$

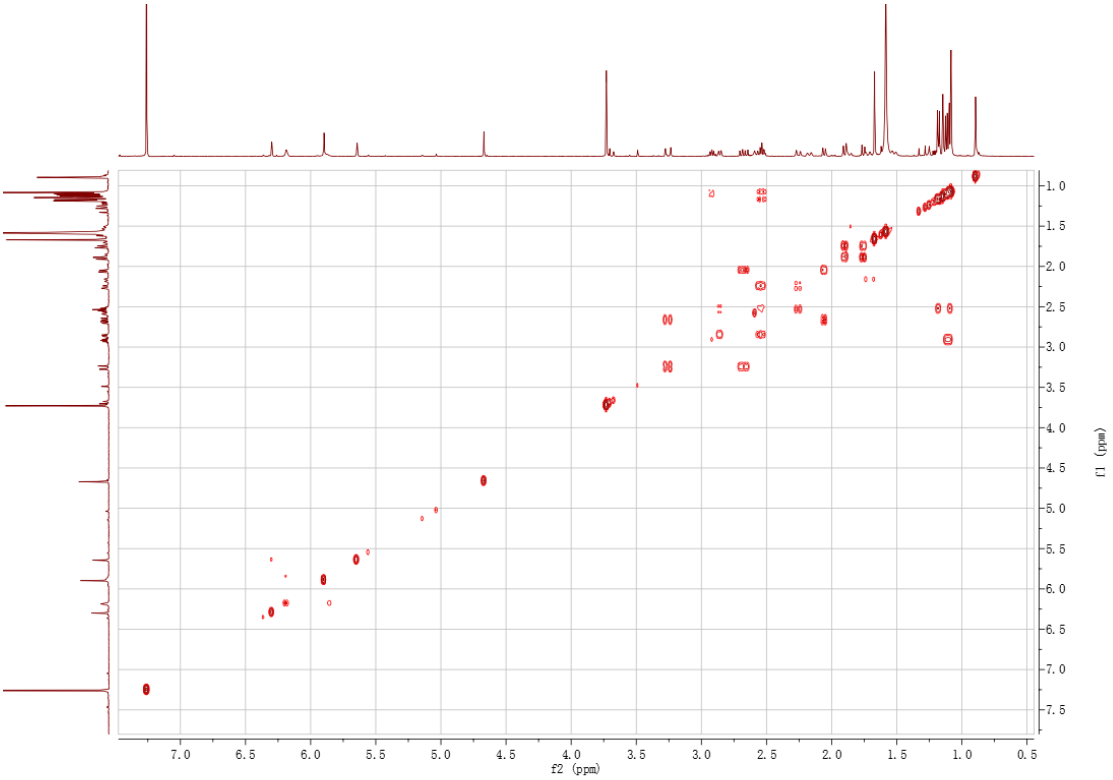

Figure S33. HMBC spectrum of compound **5** in $CDCl_3$

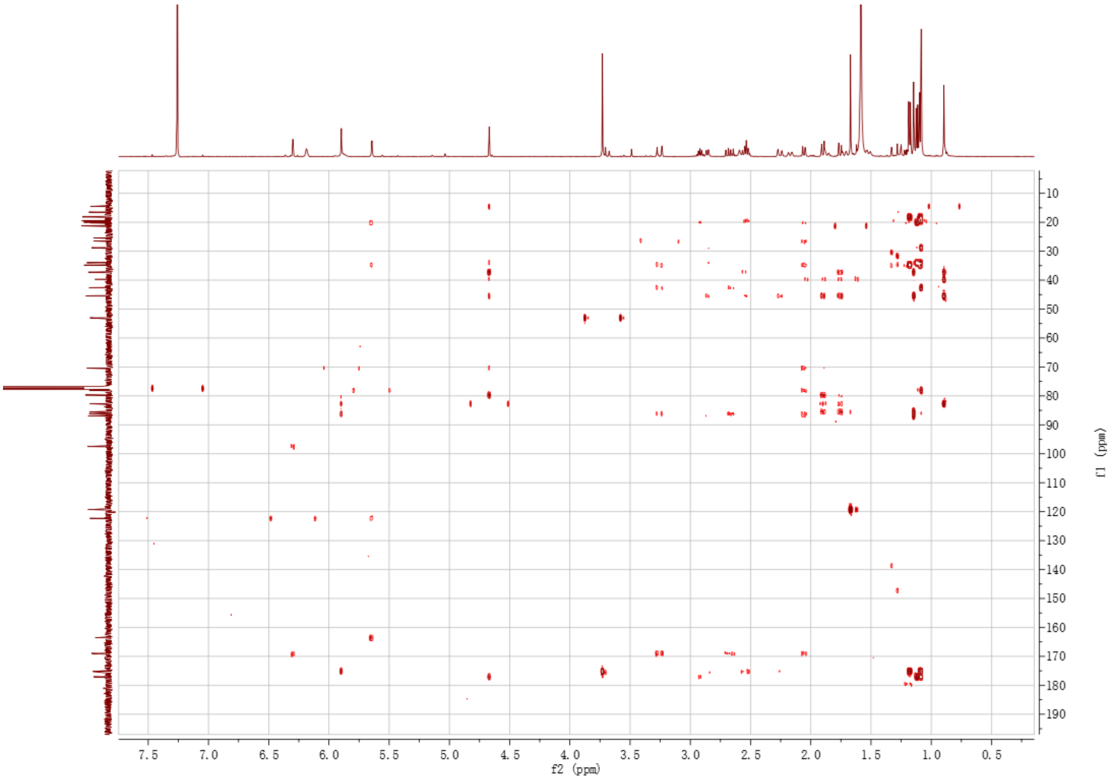

Figure S34. ROESY spectrum of compound **5** in $CDCl_3$

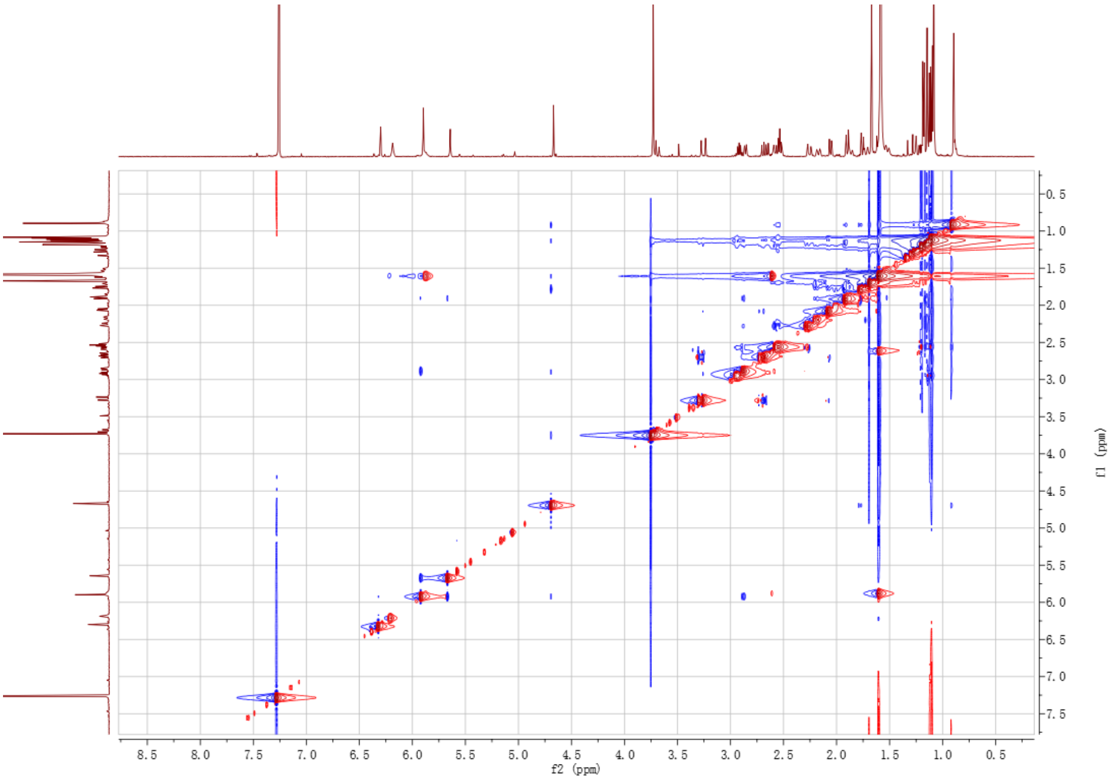

Figure S35. HRESIMS spectrum of compound **5**

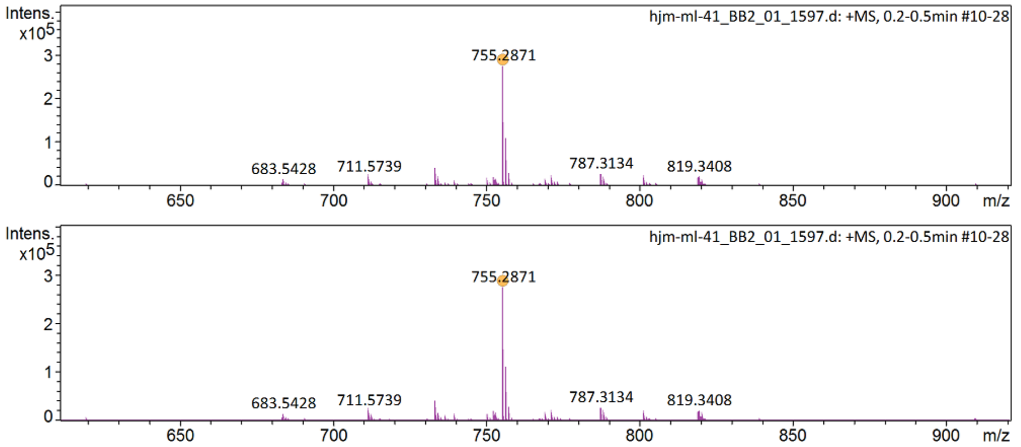

Meas. m/z	#	Ion Formula	m/z	err [ppm]	mSigma	# mSigma	Score	rdb	e¯ Conf	N-Rule	Adduct
755.2871	1	C37H48NaO15	755.2885	2.0	7.3	1	100.00	14.0	even	ok	M+Na
